# Sanggenol L Enhances Temozolomide Drug Sensitivity by Inhibiting Mitophagy and Inducing Apoptosis Through the Regulation of the TRIM16‐OPTN Axis in Glioblastoma

**DOI:** 10.1002/advs.202502915

**Published:** 2025-09-24

**Authors:** Hongbo Chang, Jianbing Hou, Xin Hu, Nana Hou, Minghao Xu, Yi Du, Jingyang Xu, Yongzhao Wang, Zhuohao Xie, Junbo Shi, Yundong Zhang, Hongjuan Cui

**Affiliations:** ^1^ State Key Laboratory of Resource Insects Medical Research Institute Southwest University Chongqing 400715 China; ^2^ Jinfeng Laboratory Chongqing 401329 China; ^3^ Department of Neurology the Third Affiliated Hospital of Chongqing Medical University Chongqing 401120 China

**Keywords:** autophagy, glioblastoma, OPTN, Sanggenol L, temozolomide, TRIM16

## Abstract

Glioblastoma (GBM) is the most aggressive and lethal form of glioma, with current standard‐of‐care treatments including surgical resection, radiotherapy, and chemotherapy with temozolomide (TMZ). However, therapeutic resistance to TMZ frequently arises, partly attributed to autophagy, as demonstrated by analysis of clinical glioblastoma specimens. Through screening of mulberry metabolites, a bioactive small molecule, Sanggenol L (SL) is identified, which inhibits glioblastoma growth and blocks autophagy flux, thereby markedly enhancing TMZ chemosensitivity when delivered via a liposome‐based system. Mechanistically, SL is found to suppress mitophagy by promoting ubiquitin‐mediated proteasomal degradation of OPTN. Moreover, the first evidence that SL upregulates TRIM16 expression is presented, which acts as an E3 ubiquitin ligase for OPTN degradation in glioblastoma. TRIM16 depletion or OPTN overexpression partially abrogated SL‐induced suppression of autophagy and apoptosis in GBM cells. Collectively, these findings suggest that SL enhances TMZ sensitivity by disrupting autophagy and inducing apoptosis through TRIM16‐mediated OPTN degradation.

## Graphical abstract

Sanggenol L, a flavonoid from mulberry, enhances glioblastoma sensitivity to temozolomide by inhibiting mitophagy and inducing apoptosis through TRIM16‐mediated OPTN degradation. In animal studies, liposomal delivery of Sanggenol L significantly improves the efficacy of its combination with TMZ, providing new insights into glioblastoma treatment.

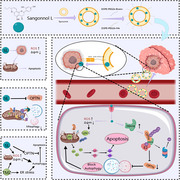

## 1 Introduction

Gliomas are the most prevalent and deadly primary tumors of the central nervous system, comprising 28% of all primary brain tumors and 80% of brain malignancies. Arising from glial stem or progenitor cells, gliomas are graded from 1 to 4 based on their malignancy, in accordance with the 2021 WHO Classification of Tumors of the Central Nervous System.^[^ ^1^ ^]^ Glioblastoma multiforme (GBM), the most aggressive and high‐grade subtype of diffuse glioma, is characterized by extensive infiltration and rapid progression. GBM accounts for ≈46.6% of all adult central nervous system malignancies.^[^ ^2^ ^]^ The incidence of GBM increases with age, with the median age of diagnosis being 64 years.^[^ ^3^ ^]^ Despite aggressive treatment involving maximal surgical resection followed by radiotherapy and chemotherapy, a definitive cure remains elusive. The median overall survival is ≈14 months, while the 5‐year survival rate is a dismal 5.5%.^[^ ^4^ ^]^

In recent years, increasing attention has been given to the efficacy of chemotherapy drugs in treating malignant gliomas. Nevertheless, the prognosis for patients undergoing temozolomide (TMZ) treatment remains dismal, with median survival ranging from 12.1 to 14.6 months. In elderly patients with glioblastoma, survival declines even further, frequently falling below 10 months.^[^ ^5^, ^6^ ^]^ The effectiveness of TMZ in glioblastoma multiforme (GBM) is influenced by multiple factors. TMZ primarily functions by methylating the DNA of glioma cells. However, several DNA repair mechanisms counteract this damage, including O6‐methylguanine DNA methyltransferase (MGMT), base excision repair (BER), and mismatch repair (MMR) pathways.^[^ ^7^ ^]^ These pathways contribute significantly to the resistance of gliomas to TMZ. Moreover, the autophagy‐lysosome‐dependent protein degradation pathway has been implicated in mediating TMZ resistance.^[^ ^8^ ^]^ Although certain drugs targeting these resistance mechanisms have demonstrated limited efficacy in mitigating drug resistance, they have failed to yield improvements in overall survival among GBM patients. Recent studies indicate that anticancer therapies, including TMZ, can induce autophagy, a cellular process increasingly recognized as a contributor to chemoresistance in various cancers.^[^ ^9^, ^10^, ^11^, ^12^ ^]^ Autophagy is a highly conserved biological process observed in nearly all eukaryotic cells, involved in diverse physiological and pathological processes.^[^ ^13^ ^]^ TMZ‐induced autophagy exhibits a dual role: in certain contexts, it delays cell death,^[^ ^14^, ^15^, ^16^ ^]^ whereas in others, it promotes apoptotic death in GBM cells exposed to TMZ treatment.^[^ ^17^ ^]^ Modulating autophagy in response to TMZ therapy represents a promising strategy for overcoming chemoresistance and enhancing therapeutic efficacy in GBM patients. However, the precise role of autophagy in regulating GBM cell fate remains a topic of active debate. Therefore, there is an urgent need for in‐depth research aimed at developing more effective adjuvant treatment strategies and discovering less toxic yet more efficacious approaches to overcome drug resistance.

Morus alba L., commonly known as mulberry or “sang shu,” is a plant species indigenous to China. It is widely cultivated and has been naturalized in numerous countries, where it is regarded as a valuable medical herbal.^[^ ^18^ ^]^ Recognized as a cornerstone of traditional Chinese medicine, it exhibits a wide range of pharmacological health benefits, including antioxidant, anti‐hypercholesterolemic, anti‐atherosclerosis, anti‐obesity, anti‐hyperglycemic, immunomodulatory, hypolipidemic, neuroprotective, and hepatoprotective effects. These therapeutic effects are attributed to its diverse repertoire of bioactive compounds, such as flavonoids, anthocyanins, phenolic acids, flavonols, and volatile aromatic compounds.^[^ ^19^, ^20^ ^]^

The root barks, leaves, and fruits of the white mulberry tree, commonly referred to as mulberry, have long been used in traditional medicine, offering a range of therapeutic properties. These include antihypertensive, hypoglycemic, anti‐anemic, diuretic, and hypolipidemic effects.^[^ ^21^ ^]^ Among its bioactive constituents, Sanggenol L is a flavonoid compound extracted from the root bark of the mulberry tree. SL has demonstrated cytotoxic and apoptotic activities in ovarian cancer cells by activating caspase signaling and inhibiting NF‐κB signaling pathways.^[^ ^22^ ^]^ Apoptosis, a fundamental cellular mechanism often triggered by natural compounds, plays a vital role in tumor suppression across various cancer type. SL has also been shown to induce apoptosis and cell cycle arrest in human prostate cancer cells by activating p53 and suppressing the PI3K/Akt/mTOR signaling pathway.^[^ ^23^ ^]^ Additionally, in melanoma cells, SL triggers apoptosis through activating the caspase cascade and other apoptosis‐inducing factors.^[^ ^24^ ^]^ Despite these promising findings, the anti‐cancer effects of Sanggenol L, particularly its biological role and mechanistic basis in glioblastoma, remain poorly understood. Further investigations are warranted to elucidate its therapeutic potential in GBM and other malignancies.

In this study, we screened 54 common active monomers with reported anti‐tumor activity from 936 mulberry‐derived metabolites identified through literature mining, and identified Sanggenol L as a candidate compound capable of inhibiting glioblastoma cell proliferation without damaging normal glial cell viability.^[^ ^25^ ^]^ Additionally, SL was found to suppress autophagy in glioblastoma. We further explored and clarified the underlying anticancer mechanisms of SL in glioblastoma. Our findings demonstrate that SL achieves its effects by promoting TRIM16‐mediated degradation of the OPTN protein, thereby inhibiting autophagy and inducing apoptosis.

Sanggenol L is a small molecular compound characterized by high lipophilicity. Compounds with such properties can traverse the lipid bilayer of cell membranes and penetrate the blood–brain barrier (BBB) via passive diffusion. However, such compounds often exhibit poor aqueous solubility. Therefore, it is crucial to develop a strategy that enhances solubility while maintaining efficient BBB permeability. Nanomaterials, as drug carriers, possess many unique advantages that have enabled their extensive application in drug delivery systems.^[^ ^26^, ^27^, ^28^ ^]^ Liposomes, as versatile nanocarriers, have demonstrated substantial promise in drug delivery and in vivo bioimaging, particularly for facilitating BBB transport.^[^ ^29^ ^]^ Due to their amphiphilic nature, liposomes can encapsulate both hydrophilic and lipophilic drugs. These molecules can be incorporated into the liposomes through various mechanisms: by localizing within the hydrophilic core, embedding in the hydrophobic bilayer, or conjugating to the surface of the phospholipid bilayer. This versatility makes liposomes an excellent tool for trans‐BBB drug delivery. Furthermore, liposome surface modification can prolong systemic circulation and enhance the enhanced permeability and retention (EPR) effect, thereby enabling tumor targeted drug delivery. Such modifications improve the biodistribution of drugs while minimizing off‐target toxicity.^[^ ^30^ ^]^ When employed as a delivery system for SL, liposomes have demonstrated promising outcomes. In vivo experiments in murine models and in vitro cellular studies revealed that liposomal delivery of SL significantly enhanced the chemosensitivity of TMZ‐resistant glioblastoma strains to TMZ, supporting its potential as a promising anticancer candidate.

## 2 Results

### 2.1 Sanggenol L Promotes Autophagosome Formation While Blocking Autophagic Flux, and Induces Apoptosis in Glioblastoma Cells

**Figure 1:**
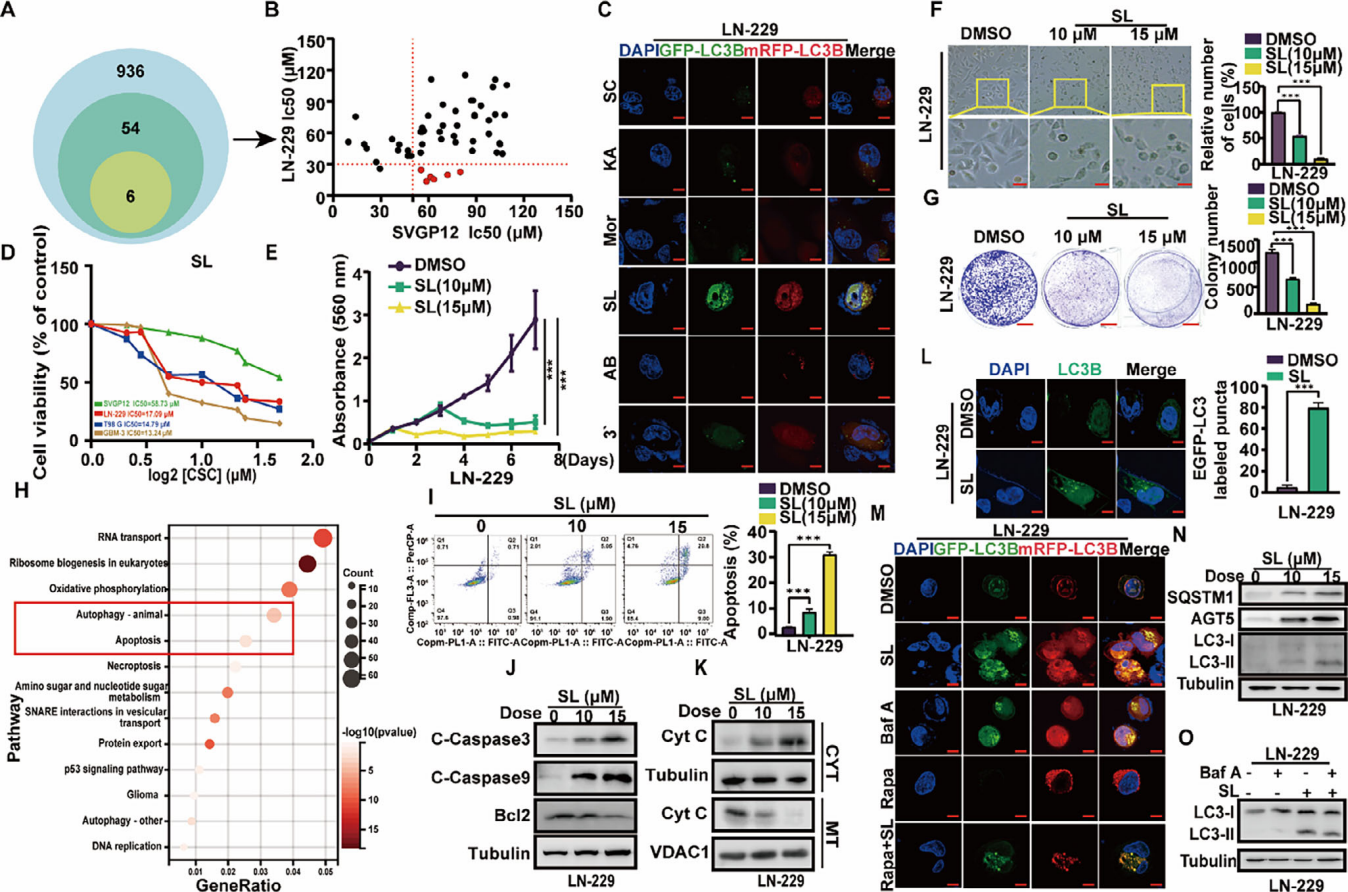
Sanggenol L blocks autophagy flux in glioblastoma and induces apoptosis of glioblastoma cells. A) Schematic diagram depicting the drug screening process. **B**) MTT assay results were used to determine the IC_50_ values of various mulberry‐derived compounds in SVGP12 and LN‐229 cell lines. **C**) Detection of autophagy in LN‐229 cells following treatment with six different mulberry compounds, examined via LC3B double‐labeling experiments. (Sanggenon C: SC; Kuwanon A: KA; Morusinol: Mor; Sanggenol L: SL; Albanol b: AB; PSX003: 3′) **D**) Treatment of GBM cells (GBM‐3, LN‐229, and T98G) and normal glial SVGP12 cells with a series of graded SL concentrations for two days. Cell viability was assessed using MTT assays, and the IC50 values of SL in the tested cells are indicated. DMSO served as the control. **E**) Viability of LN‐229 cells after treatment with SL at 10 and 15 µm. DMSO was used as the control. **F**) Morphological changes in LN‐229 cells after incubation with different concentrations of SL or DMSO for 48 h. Scale bars = 10 µm. DMSO served as the control. **G**) Evaluation of in vitro colony formation in LN‐229 cells after treatment with various concentrations of SL or DMSO. H) Orthotopic implantation was performed using GBM‐3 cells. Following the establishment of orthotopic tumors, mice were treated with SL therapy, while DMSO served as the control. Post‐treatment, blood was collected from the eye, and mouse survival was recorded. **H**) KEGG analysis of significantly altered genes in LN‐229 cells treated with SL, identified through quantitative proteomics data. The top 13 KEGG pathways, ranked by fold enrichment, are presented. DMSO served as the control. The color bar on the right represents ‐log10 (*p*‐value). **I**) Apoptosis in LN‐229 cells treated with SL (10 and 15 µm) or DMSO for two days, as detected by flow cytometry. DMSO served as the control. **J,K**) Western blot assays were conducted to assess apoptosis‐related proteins, including Bcl‐2, C‐Caspase3, C‐Caspase9, and Cytochrome C (cytoplasmic and mitochondrial fractions), in LN‐229 cells treated with specified SL concentrations and durations for two days. DMSO was used as the control. **L)** Immunofluorescence staining of LC3B (green) in LN‐229 cells after treatment with or without SL (10 µm) for two days. Nuclei were counter‐stained with DAPI (blue). Scale bars = 10 µm. **M**) Immunofluorescence analysis of LN‐229 cell transiently transfected with mRFP‐EGFP‐LC3, followed by treatment with DMSO, rapamycin (Rapa,  nm), chloroquine (CQ, 10 µm), SL (10 µm), or a combination of Rapa (1 nm) and SL (10 µm). Scale bars = 20 µm. Cells treated with rapamycin and CQ were used as positive and negative controls, respectively. **N**) Western blot analysis of autophagy‐related proteins, including SQSTM1, ATG5, and LC3B, in LN‐229 cell treated with specified concentrations and durations of SL for two days. DMSO served as the control. **O**) Expression of LC3B in LN‐229 cell following treatment with SL and bafilomycin A1 (Baf A), detected via Western blot (WB). Data are expressed as mean ± SD. Statistical significance was determined using Student's t‐test. ^*^ *p* < 0.05, ^**^ *p* < 0.01, ^***^ *p* < 0.001.

Immunohistochemical staining revealed that LC3B and ATG5 expression levels increased with glioblastoma grade, whereas cleaved caspase‐3 (C‐Caspase3) expression decreased. A similar trend was observed in patients receiving TMZ chemotherapy: LC3B and ATG5 expression levels were elevated, while C‐Caspase3 expression levels were reduced (Figure S1, Supporting Information). Through literature retrieval, 54 monomers with anti‐tumor activity were identified among the 936 metabolites of mulberry trees (Table S1, Supporting Information). MTT assays demonstrated that 6 of 54 compounds exhibited significantly higher IC_50_ values in normal glial cells (SVGP12) compared to glioblastoma cells (LN‐229) (**Figure** **1**A,B). Following 24 h treatments with each of these six candidates, LC3B double‐labeling assays revealed that one compound, Sanggenol L, markedly inhibited autophagic flux in glioblastoma cells (Figure 1C). To expand the screening for agents capable of reducing TMZ resistance in glioblastoma, we examined the effects of eight compounds (with IC50 values below 30 µM in LN‐229 cells) on MGMT expression in MGMT‐overexpressing HEK293FT cells. Fluorescent reporter assay results showed that Sanggenol L and compound Albanol B reduced MGMT expression in HEK293FT cells. Additionally, Western blot (WB) analysis confirmed that compounds SL and AB decreased MGMT expression in T98G and GBM‐3 cells (note: the LN‐229 cell line could not be tested due to MGMT methylation^[^ ^31^ ^]^) (Figure S2, Supporting Information). The structural makeup of SL is provided in Figure S2 (Supporting Information). To further evaluate the antitumor potential of SL, GBM‐3, LN‐229, T98G, and normal glial SVGP12 cells were treated with varying concentrations of SL for 48 h. Results showed that even at relatively low concentrations, SL markedly inhibited cell growth. Notably, the median lethal concentration for SVGP12 cells was higher than that for GBM‐3, LN‐229, and T98G cells (Figure 1D). Appropriate concentrations of SL (10 and 15 µM) were then administered to GBM‐3, LN‐229, and T98G cells, with DMSO serving as a control, for 48 h. MTT assays confirmed that SL significantly suppressed the proliferation of GBM cell lines in a dose‐dependent manner (Figure 1E; Figure S4A, Supporting Information). Microscopy further illustrated a marked decrease in viable GBM cells with increasing SL concentrations. (Figure 1F; Figure S4B, Supporting Information). Further findings from an in vitro colony formation assay indicated that colony‐forming capability in the SL‐treated groups was notably diminished compared to the control group (Figure 1G; Figure S4C, Supporting Information). Quantitative proteomics‐based enrichment analysis highlighted significant changes in autophagy‐ and apoptosis‐related pathways following SL treatment (Figure 1H). Flow cytometry analysis demonstrated a significant increase in apoptotic cell populations after SL treatment. (Figure 1I; Figure S5A, Supporting Information). Consistent with these results, TUNEL staining revealed clear evidence of SL‐induced apoptosis (Figure S5B, Supporting Information). Protein analysis related to apoptosis, including cleaved caspase‐9 (C‐Caspase9), cleaved caspase‐3 (C‐Caspase3), and BCL‐2, further supported these findings. Western blot analysis of apoptosis‐related proteins showed increased levels of C‐Caspase3, C‐Caspase9, and decreased BCL‐2 protein levels in a concentration‐dependent manner. The results of the Western blot after the separation of mitochondria and cytoplasm indicate that with the increase of SL concentration, the level of cytochrome C in the mitochondria decreases significantly, while the level of cytochrome C in the cytoplasm increases significantly. This suggests that there is an outflow of apoptotic factors following mitochondrial damage. (Figure 1J,K; Figure S5C,D, Supporting Information). Autophagy, also known as type II programmed cell death, plays a crucial role in the progression of various diseases, with a complex interplay observed between autophagy and apoptosis. LC3B immunofluorescence results revealed that autophagosomes were significantly enriched in glioblastoma cells treated with SL compared to controls (Figure 1L; Figure S6A, Supporting Information). Additionally, LC3B double‐labeling adenovirus assays confirmed that SL‐induced autophagosome accumulation resulted in autophagic flux blockage in glioblastoma cells (Figure 1M; Figures S6B and S7, Supporting Information). Western blot results further corroborated the increased expression of autophagy markers LC3‐II and the pro‐autophagic effector ATG5 in GBM‐3, LN‐229, and T98G cells in a dose‐dependent manner following SL treatment (Figure 1N; Figure S6C, Supporting Information). The upregulation of ATG5, a key regulator of autophagosome biogenesis, further supports that SL promotes autophagosome formation. However, the simultaneous accumulation of p62 indicates that the autophagic process cannot be completed—likely due to impaired fusion between autophagosomes and lysosomes or defective lysosomal degradation function—resulting in blocked autophagic flux. Collectively, these findings suggest that SL can stimulate the formation of autophagosomes in glioma cells. To further investigate SL's effect on autophagy in glioblastoma, LC3‐II expression was analyzed under SL treatment with or without bafilomycin A1 intervention. The absence of further LC3‐II accumulation under these conditions suggested that SL may heighten LC3‐II levels by inhibiting autophagy flux (Figure 1O; Figure S6D, Supporting Information). These results collectively demonstrate that SL does not simply “activate autophagy” in a general sense, but rather specifically promotes autophagosome formation while blocking late‐stage autophagic flux. This disruption of autophagic integrity prevents cells from effectively clearing damaged organelles and protein aggregates via autophagy, leading to increased cellular stress and ultimately inducing apoptosis, which is consistent with our observed apoptotic phenotypes.

### 2.2 Sanggenol L Degrades the Autophagy Receptor OPTN by Ubiquitination, Causing Mitochondrial Autophagy Blockade

**Figure 2:**
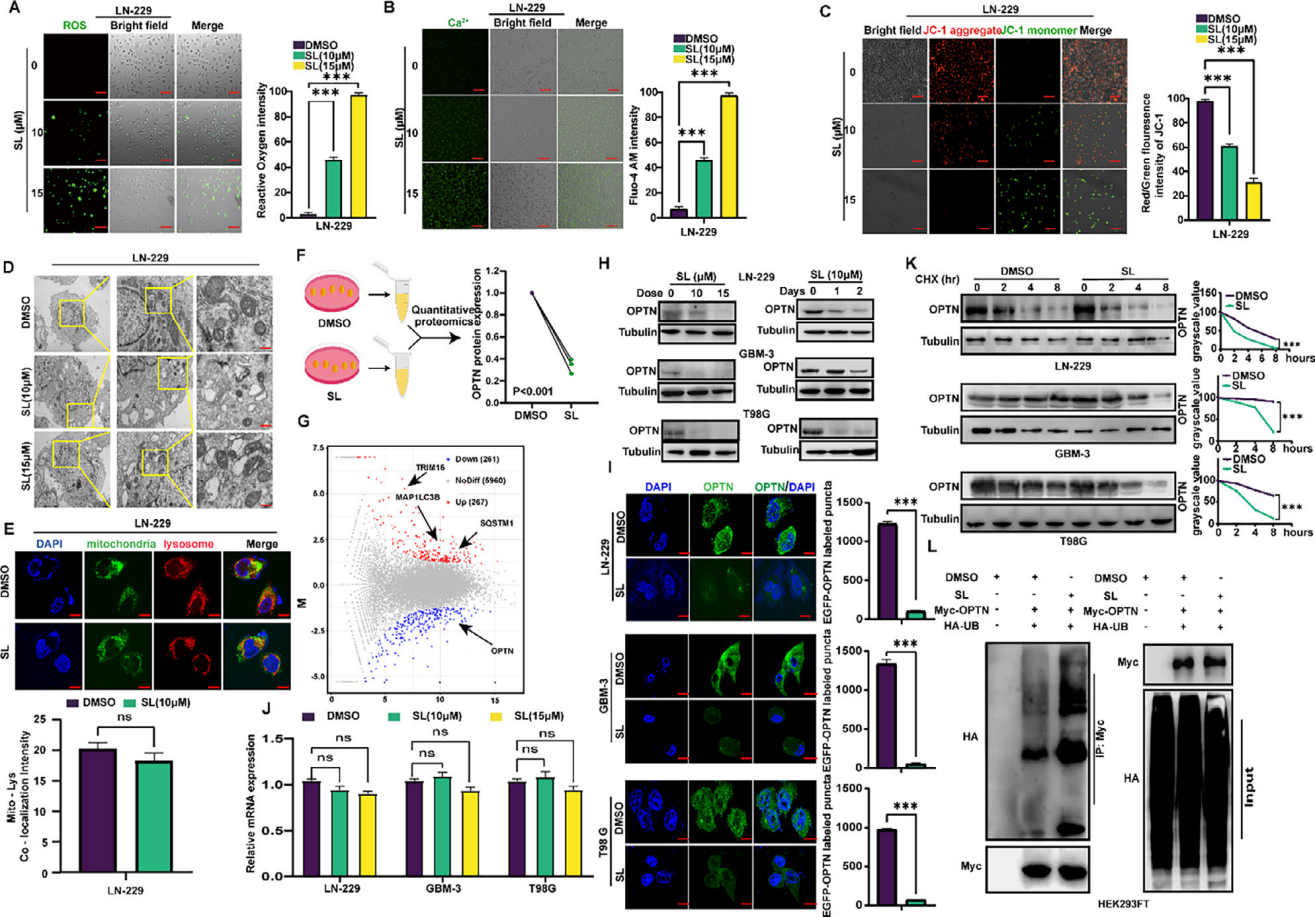
Sanggenol L degrades the autophagy receptor OPTN by ubiquitination, causing mitochondrial autophagy blockade. **A**) Fluorescence imaging and statistical analysis of cells stained with the Reactive Oxygen Species Assay after treatment with SL (10 and 15 µm). DMSO was used as the control. **B**) Fluorescence imaging and statistical analysis of cells stained with the Fluo‐4 AM calcium ion fluorescent probe after treatment with SL (10 and 15 µm). DMSO was used as the control. **C**) Fluorescence imaging and statistical analysis of cells stained with JC‐1 after treatment with SL (10 and 15 µm). Red fluorescence indicates mitochondrial aggregates, while green fluorescence represents mitochondrial monomers. Scale bar: 50 µm. **D**) Electron microscopy images of glioblastoma cell line LN‐229 treated with 10 µm SL for 12 h following autophagy induction. DMSO was used as the control. Scale bar: 50 µm. **E**) Fluorescence images of mitochondrial and lysosomal staining in glioblastoma cells (LN‐229) treated with 10 µm SL for 12 h. Green fluorescence represents mitochondria, and red fluorescence represents lysosomes. DMSO was used as the control. Scale bar: 50 µm. **F**) Quantitative proteomics analysis of LN‐229 cells after treatment with SL (10 µm). DMSO was used as the control. **G**) Quantitative proteomics analysis of LN‐229 cells after treatment with SL (10 µm), presented as a volcano plot to visualize the number of upregulated and downregulated proteins. **H**) Western blot assays were performed to assess the protein expression level of OPTN in GBM‐3, LN‐229, and T98G cells after treatment with SL (10 and 15 µm) or DMSO for 2 days. DMSO was used as the control. **I**) Immunofluorescence analysis of OPTN in GBM‐3, LN‐229, and T98G cells treated with SL (10 µm) or DMSO. DMSO was used as the control. Scale bar: 20 µm. **J**) RT‐PCR assays were performed to evaluate the mRNA expression level of OPTN in GBM‐3, LN‐229, and T98G cells after treatment with the indicated concentrations of SL for 2 days. DMSO was used as the control. **K**) GBM‐3, LN‐229, and T98G cells were treated with SL (10 µm) or DMSO, followed by treatment with CHX (100 µg mL^−1^) for the indicated times. Cells were then harvested, and the OPTN turnover rate was analyzed through Western blot analysis. Grayscale values were included on the WB bands, with 0 h used as the baseline for each group. DMSO served as the control. **L**) HEK293FT cells transfected with the indicated plasmids were treated with SL (10 µm) or DMSO. Ubiquitination of OPTN was subsequently detected through Western blot analysis. DMSO was used as the control. The data were expressed as mean ± SD. Student's t‐test was performed to analyze statistical significance. ^*^ *p* < 0.05, ^**^ *p* < 0.01, ^***^ *p *< 0.001.

SL treatment led to a marked increase in reactive oxygen species (ROS) levels in glioblastoma cells (**Figure** **2**A). This was accompanied by a significant rise in intracellular calcium ions and a decrease in mitochondrial membrane potential, both of which correlated positively with elevated SL concentration and prolonged exposure duration (Figure 2A–C; Figure S8A–C, Supporting Information). Transmission electron microscopy (TEM) images and immunofluorescence colocalization of mitochondria and lysosomes were performed on glioblastoma cells treated with 15 µm SL for 12 h (Figure 2D,E). These observations indicate that, although SL induces autophagosome accumulation in glioblastoma cells, mitochondria failed to fuse with autolysosomes. As shown in Figure 2F,G (Supporting Information), gene enrichment analysis revealed a significant association with autophagy following SL treatment. Quantitative proteomic profiling further revealed substantial downregulation of the mitochondrial autophagy receptor OPTN. To further investigate whether SL affects other autophagy receptors. We performed proteomics analysis on SL‐treated samples and used Western blot to detect the expression levels of other autophagy receptors after SL treatment. The results showed that SL primarily affects the autophagy process by regulating the expression of OPTN, while its impact on other autophagy receptors is relatively minor (Figure S9, Supporting Information). Consistently, Western blot analysis confirmed that SL reduced OPTN protein level in a time‐ and concentration‐dependent manner in glioblastoma cell lines GBM‐3, LN‐229, and T98G (Figure 2). Immunofluorescence imaging further revealed pronounced degradation of OPTN in glioblastoma cells after SL treatment (Figure 2I). To explore the mechanism underlying SL‐mediated regulation of OPTN, we performed reverse transcription polymerase chain reaction (RT‐PCR) assays. Results showed that SL did not affect the mRNA expression levels of OPTN (Figure 2J). Based on these observations, we hypothesized that SL modulates OPTN protein stability post‐translationally. Notably, SL appeared to modulate OPTN turnover when co‐administered with cycloheximide (CHX), an inhibitor of de novo protein synthesis (Figure 2K). To confirm SL's effect on OPTN stabilization, we assessed the ubiquitination levels of OPTN. Results showed that SL treatment significantly increased OPTN ubiquitination (Figure 2L).

### 2.3 Sanggenol L Causes Autophagy Blockade and Apoptosis of GBM Cells by Inhibits OPTN Expression

**Figure 3:**
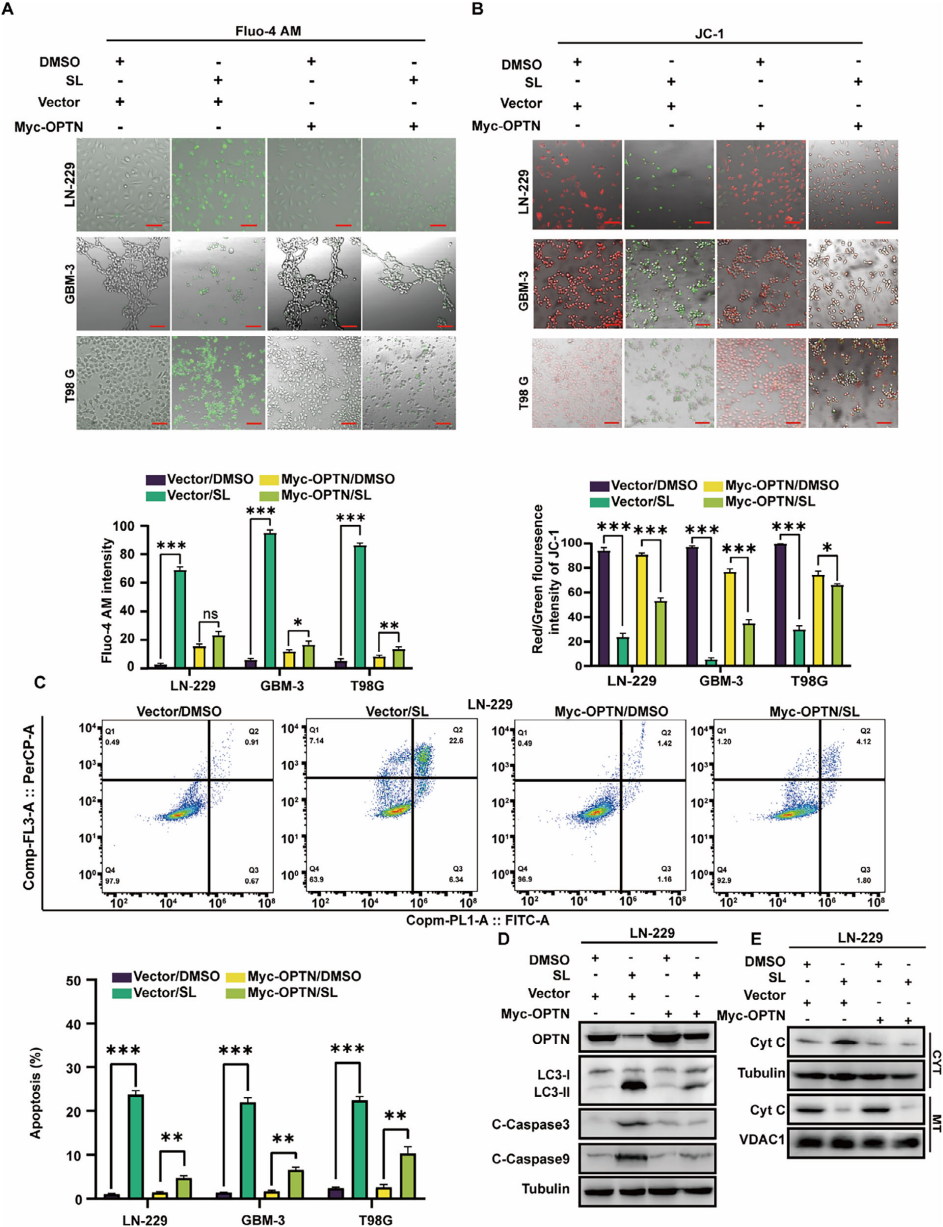
Overexpression of OPTN can partially restore the effects of Sanggenol L on GBM. A) Fluorescence imaging and statistical analysis of cells stained with the Fluo‐4 AM calcium ion fluorescent probe after treatment with SL (10 and 15 µm). DMSO was used as the control. **B**) Fluorescence imaging and statistical analysis of cells stained with JC‐1 after treatment with SL (10 and 15 µm). DMSO served as the control. (Red: mitochondrial aggregates; green: mitochondrial monomers). Scale bar: 50 µm. **C**) Apoptosis in GBM‐3, LN‐229, and T98G cells treated with SL (10 and 15 µm) or DMSO for 2 days was analyzed via flow cytometry. DMSO was used as the control. **D,E**) Western blot assays were conducted to evaluate apoptosis‐related proteins, including Bcl‐2, C‐Caspase3, C‐Caspase9, and Cytochrome C (in the cytoplasm or mitochondria), in GBM‐3, LN‐229, and T98G cells after treatment with the indicated concentrations of SL for 2 days. DMSO was used as the control. The data were expressed as mean±SD. Student's t‐test was performed to analyze significance. ^*^ *p* < 0.05, ^**^ *p* < 0.01, ^***^ *p* < 0.001.

We hypothesized that OPTN plays a crucial role in the SL‐induced autophagy inhibition and apoptosis induction in glioblastoma cells. To investigate this, we established stable OPTN‐overexpressing cell lines via lentiviral transduction. Intracellular calcium measurement and mitochondrial membrane potential assays demonstrated that OPTN overexpression markedly mitigated SL‐induced mitochondrial damage (**Figure** **3**A,B; Figure S10A,B, Supporting Information). Furthermore, flow cytometry analysis revealed that OPTN overexpression significantly reduced SL‐induced apoptosis in glioblastoma cells (Figure 3C; Figure S11A, Supporting Information). Western blot analysis corroborated these findings, showing that OPTN overexpression reduced the protein levels of OPTN, LC3BII/I, cleaved Caspase‐9, and cleaved Caspase‐3 in SL‐treated GBM cells (Figure 3D; Figure S11B, Supporting Information). Consistently, mitochondrial cytoplasmic isolation assays further confirmed that OPTN overexpression substantially inhibited the release of cytochrome C, a key mitochondrial apoptosis factor triggered by SL (Figure 3E; Figure S11C, Supporting Information).

### 2.4 Sanggenol L Decreases the Protein Stability of OPTN Through TRIM16

**Figure 4:**
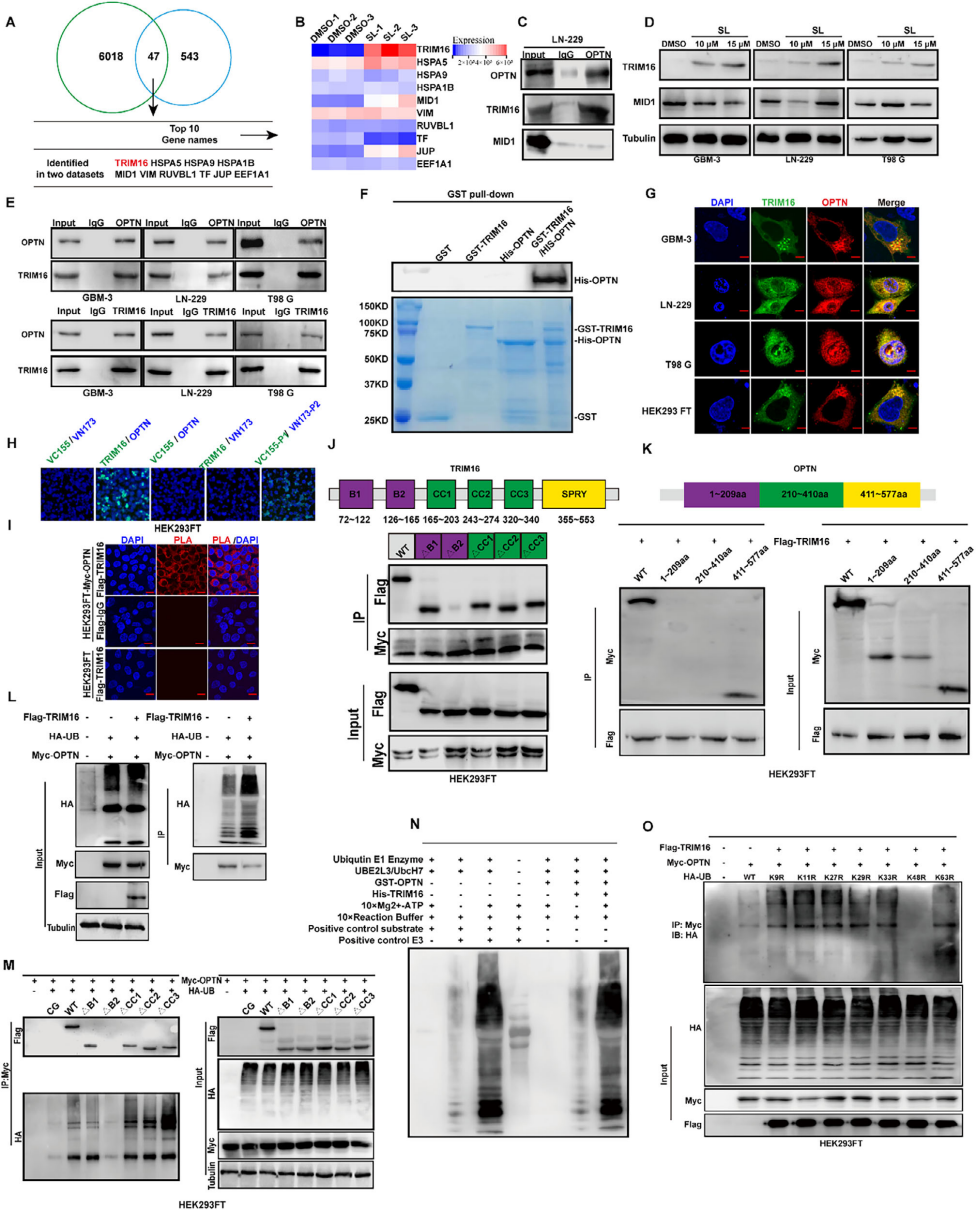
TRIM16 specifically binds to OPTN and acts as an E3 ubiquitin ligase to degrade OPTN. A) Overexpression of OPTN in HEK293 cells, followed by co‐immunoprecipitation (Co‐IP) and mass spectrometry analysis, along with quantitative proteomic profiling of LN‐229 cells treated with SL (10 µm). The combination of mass spectrometry and quantitative proteomics was utilized for this analysis. B) Proteomic and mass spectrometry analysis of glioblastoma samples after SL treatment, resulting in a heatmap that highlights the expression of the top ten proteins. C) Co‐immunoprecipitation (Co‐IP) was used to confirm the interaction between OPTN and TRIM16, as well as MID1. D) Western blot (WB) analysis was conducted to examine the expression levels of TRIM16 and MID1 in GBM‐3, LN‐229, and T98G cells after SL treatment. E) Overexpression of OPTN in GBM‐3, LN‐229, and T98G cells, followed by Co‐IP and Western blot analysis. F) A GST pull‐down assay demonstrated the direct interaction between OPTN and TRIM16. G) Immunofluorescence colocalization assay confirmed the direct interaction between OPTN and TRIM16 within GBM cells. (Red: TRIM16; Green: OPTN). H) Bimolecular fluorescence complementation (BiFC) assay indicated the direct interaction between OPTN and TRIM16 in GBM cells. I) DuoLink proximity ligation assay (PLA) validated the direct interaction between OPTN and TRIM16 in GBM cells. J,K) Truncation and domain deletion vectors for OPTN and TRIM16 were employed to identify the interaction domains. L) Indicated plasmids were transfected into HEK293FT cells, and OPTN ubiquitination was assessed via Western blot analysis. M) A Western blot (WB) was performed to analyze the effect of TRIM16 with missing structural domains on OPTN ubiquitination. N) WB detection of in vitro ubiquitination between TRIM16 and OPTN. O) Ubiquitination assay and Western blot were used to identify TRIM16 ubiquitination sites on OPTN. The data were expressed as mean±SD. Student's *t* test was performed to analyze significance. ^*^ *p* < 0.05, ^**^ *p* < 0.01, ^***^ *p* < 0.001.

Using Co‐IP followed by mass spectrometry and quantitative proteomic analysis after SL treatment, we identified the top ten candidate OPTN‐interacting proteins. Among these, TRIM16 and MID1 were notable for their known functions as E3 ubiquitin ligases. The quantitative proteomics heatmap data revealed a significant upregulation of TRIM16 in response to SL treatment (**Figure** **4**A,B). To further validate these interactions, we conducted co‐immunoprecipitation (Co‐IP) experiments on targeting OPTN, TRIM16, and MID1 in the LN‐229 cell line. Results demonstrated TRIM16 exhibited a stronger interaction with OPTN than MID1 (Figure 4C). Subsequently, we treated LN‐229, GBM‐3, and T98G cell lines with increasing concentrations of SL. TRIM16 expression increased in a dose‐dependent manner, while MID1 expression remained unchanged. (Figure 4D). Immunohistochemistry analysis of GBM samples revealed a negative correlation between TRIM16 and OPTN expression (Figure S12A, Supporting Information). To verify the interaction between TRIM16 and OPTN in GBM cells, we performed multiple analyses, including Co‐IP, Bimolecular fluorescence complementation (BIFC), Duo‐link proximity ligation assay (PLA), and immunofluorescence co‐localization. All methods consistently confirmed the interaction between TRIM16 and OPTN in glioblastoma cells (Figure 4E–I). We further investigated the binding domains of TRIM16 and OPTN using truncation and deletion mutants. The results indicated that the B‐BOX domain of TRIM16 interacts with the 411–577 amino acid functional domain of OPTN (Figure 4J,K; Figure S12B, Supporting Information). Ubiquitination experiments revealed that TRIM16 overexpression significantly enhanced OPTN ubiquitination (Figure 4L). In vitro ubiquitination assays further confirmed that TRIM16 functions as an E3 ubiquitin ligase targeting OPTN (Figure 4N). Notably, deletion of the TRIM16 binding domain markedly attenuated OPTN ubiquitination enhancement (Figure 4M). Moreover, ubiquitination experiments using wild‐type and mutant ubiquitin plasmids demonstrated that TRIM16 failed to increase OPTN ubiquitination in the absence of K48. These findings suggest that TRIM16 mediates K48‐linked polyubiquitination of OPTN (Figure 4O). We utilized databases including PhosphoSitePlus, UbiBrowser, and UbPred to computationally predict potential ubiquitination sites on OPTN. This analysis identified three candidate lysine residues at positions 448, 489, and 501. To validate these potential sites, we constructed expression plasmids carrying single lysine‐to‐arginine mutations (K448R, K489R, K501R) as well as an OPTN triple mutant (K448R/K489R/K501R). We then performed ubiquitination assays in HEK293FT cells by co‐expressing these mutant constructs with TRIM16. Our results showed that mutations at positions 489 and 501 significantly at positions 489 and 501 significantly reduced TRIM16‐mediated K48‐linked ubiquitination of OPTN compared to wild‐type OPTN. Notably, the ubiquitination level was most dramatically decreased in the triple mutant. These findings indicate that the lysine residues at positions 489 and 501 are critical sites for TRIM16‐mediated ubiquitination of OPTN (Figure S12C, Supporting Information). Based on these findings, we hypothesized that TRIM16 regulates OPTN protein stability. Supporting this notion, cycloheximide (CHX) chase assays demonstrated that TRIM16 promoted OPTN degradation by accelerating its turnover, suggesting post‐translational regulation. (Figure S12D, Supporting Information).

### 2.5 Sanggenol L Induces Autophagy Blockade Through the TRIM16‐OPDN Axis, Which in Turn Induces Apoptosis

**Figure 5:**
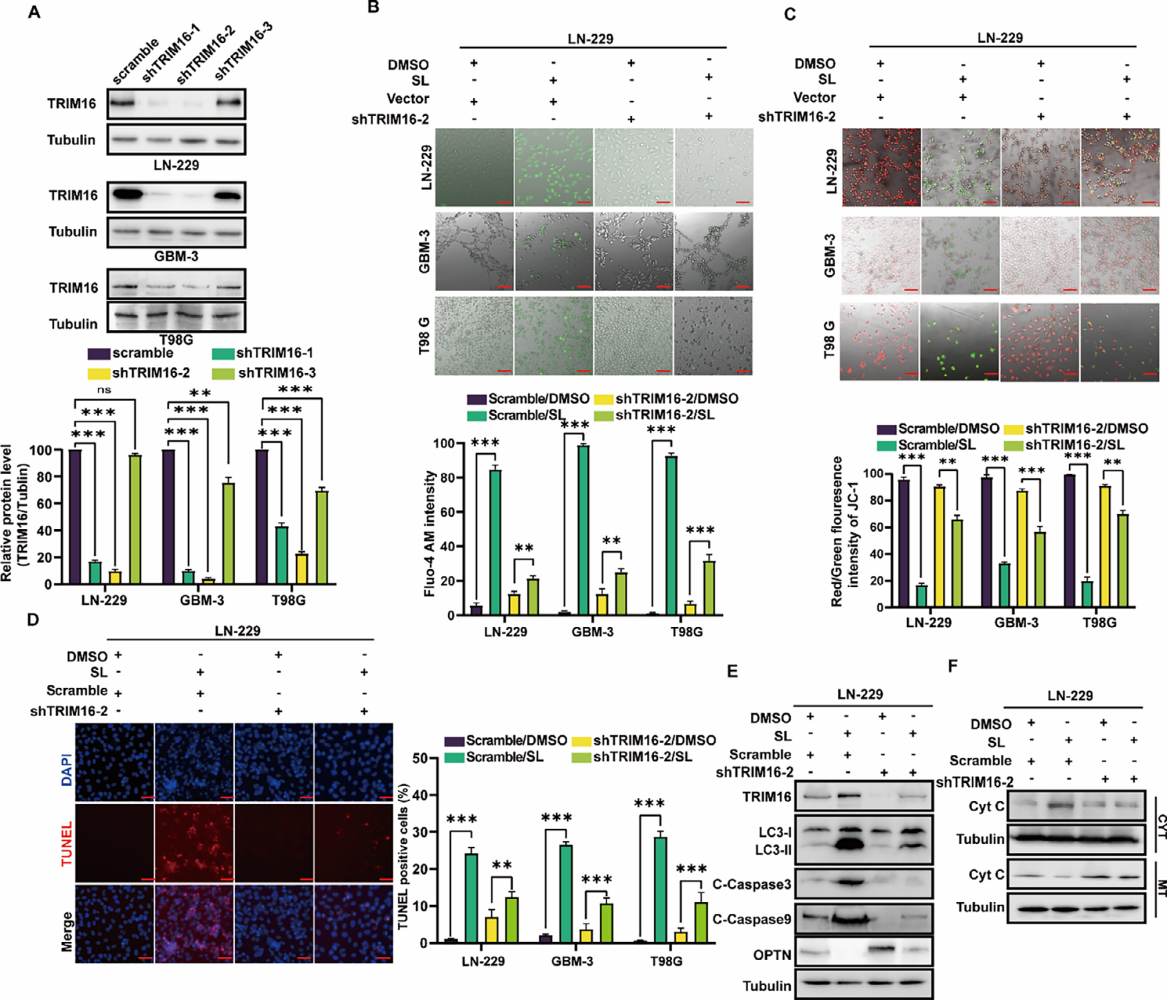
Sanggenol L decreases the protein stability of OPTN through TRIM16. A) Western blot (WB) analysis illustrating the interference efficiency of TRIM16. B) Fluorescence imaging and statistical analysis of cells stained with the Fluo‐4 AM calcium ion fluorescent probe following treatment with SL (10 and 15 µm). DMSO served as the control. C) Fluorescence imaging and statistical analysis of cells stained with JC‐1 after treatment with SL (10 and 15 µm). DMSO was used as the control. Mitochondrial aggregates are represented in red, and mitochondrial monomers are shown in green. Scale bar: 50 µm. D) Representative TUNEL staining images of GBM‐3, LN‐229, and T98G cells following treatment with SL (10 and 15 µm) or DMSO for 2 days. Scale bar: 50 µm. DMSO served as the control. E,F) Western blot assays were performed to evaluate apoptosis‐related proteins, including Bcl2, C‐Caspase3, C‐Caspase9, and Cytochrome C (in both the cytoplasm and mitochondria) in GBM‐3, LN‐229, and T98G cells. Cells were treated with the indicated SL concentrations for 2 days. DMSO was used as the control. To further confirm the effect of SL on TRIM16, we detected the expression of TRIM16 mRNA in LN‐229, T98 G, and GBM‐3 cells after SL treatment using quantitative polymerase chain reaction (qPCR). We found that the mRNA expression levels of the TRIM16 gene were upregulated in a dose‐dependent manner in all three cell lines. Subsequently, we screened for potential transcription factors of TRIM16 by combining transcriptomics with the TF‐Target Finder tool and identified six potential transcription factors, which we displayed using a heatmap. The data were expressed as mean±SD. Student's t‐test was performed to analyze significance. ^*^ *p* < 0.05, ^**^ *p* < 0.01, ^***^ *p* < 0.001.

We hypothesize that SL targets the TRIM16‐OPTN axis to block autophagy and induce apoptosis in GBM cells. To investigate the biological functions of TRIM16, we generated TRIM16‐knockdown GBM cells and selected the most efficient shRNA# 2 for subsequent experiments (**Figure** **5**A; Table S2, Supporting Information). To test this hypothesis, TRIM16‐knockdown GBM cells were treated with either SL or a DMSO control. Calcium imaging and mitochondrial membrane potential assays revealed that TRIM16 knockdown significantly reduced SL‐induced mitochondrial damage in GBM cells (Figure 5B,C; Figure S13A,B, Supporting Information). TUNEL staining assays further confirmed that TRIM16 knockdown inhibited SL‐induced apoptosis, which was attributed to reduced mitochondrial damage (Figure 5D; Figure S14A, Supporting Information). Western blot analysis demonstrated SL‐treated TRIM16‐knockdown GBM cells exhibited markedly reduced levels of OPTN, LC3B‐II/I, cleaved Caspase‐9, and cleaved Caspase‐3 (Figure 5E; Figure S14B, Supporting Information). Consistently, mitochondrial cytoplasmic isolation assays via western blotting showed that TRIM16 knockdown significantly suppressed SL‐induced release of cytochrome C, a key mitochondrial apoptosis factor (Figure 5F; Figure S14C, Supporting Information). To further confirm the effect of SL on TRIM16, we detected the expression of TRIM16 mRNA in LN‐229, T98 G, and GBM‐3 cells after SL treatment using quantitative polymerase chain reaction (qPCR). We found that the mRNA expression levels of the TRIM16 gene were upregulated in a dose‐dependent manner in all three cell lines. Subsequently, we screened for potential transcription factors of TRIM16 by combining transcriptomics with the TF‐Target Finder tool and identified six potential transcription factors, which we displayed using a heatmap (Figure S15, Supporting Information).

### 2.6 Sanggenol L Improves TMZ Chemosensitivity by Blocking Protective Autophagy of TMZ‐Resistant Strains

**Figure 6:**
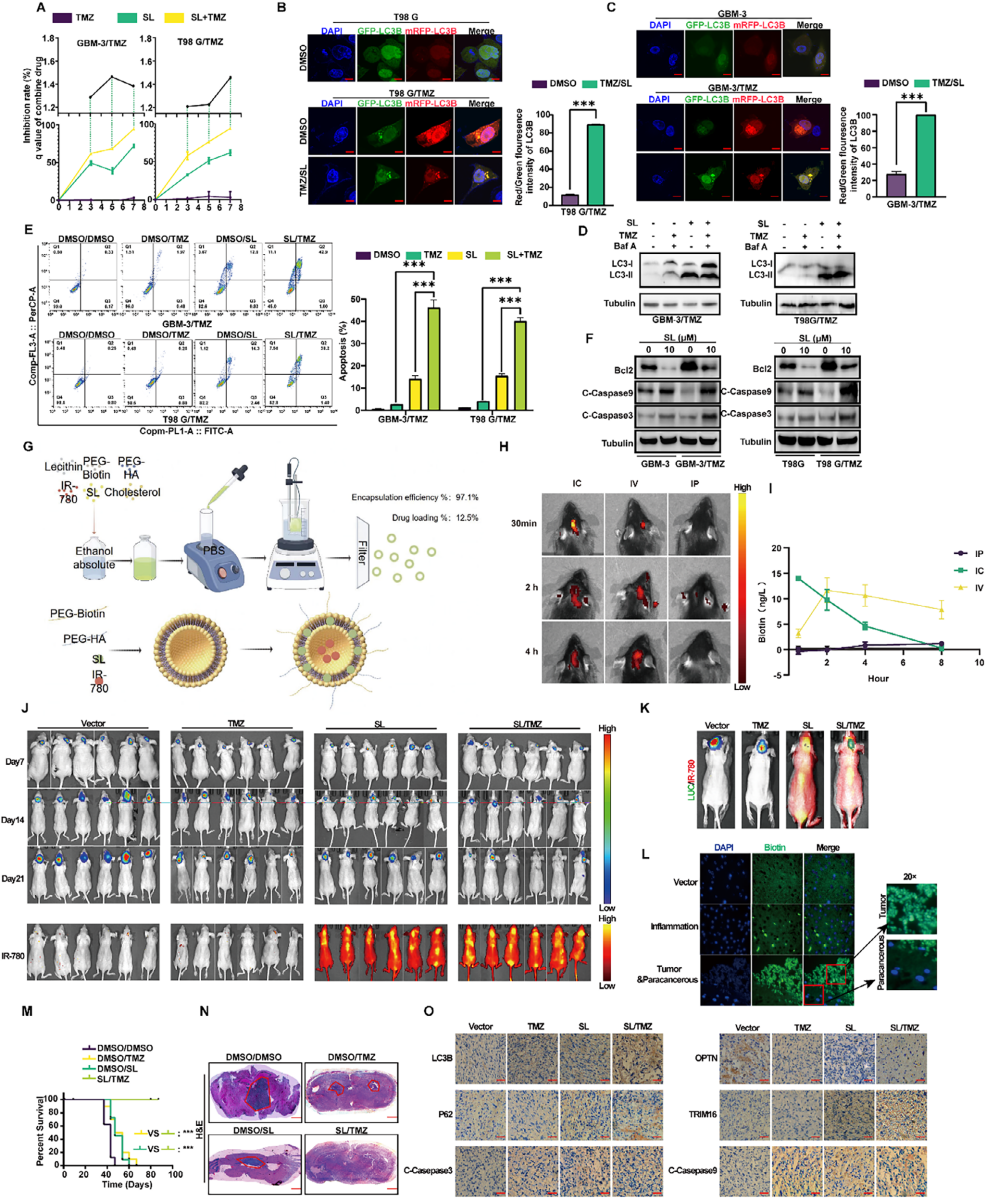
Liposomes carrying Sanggenol L can specifically accumulate at tumor sites in mice and enhance the sensitivity to TMZ. A) Cell proliferation of GBM‐3, LN‐229, and T98G cells treated with SL (10 µm), TMZ (300 µm), or a combination of SL (10 µm) and TMZ (300 µm) for the indicated days was analyzed using MTT assays. DMSO was used as a control. B,C) Immunofluorescence analysis of GBM‐3 and T98G temozolomide‐resistant cell lines transiently transfected with mRFP‐EGFP‐LC3, followed by treatment with SL (10 µm) or DMSO. DMSO served as the control. Scale bar: 20 µm. D) Western blot analysis was conducted to assess autophagy in GBM‐3 and T98G temozolomide‐resistant cell lines. E) Apoptosis in GBM‐3 and T98G temozolomide‐resistant cell lines treated with SL (10 and 15 µm) or DMSO for two days was measured via flow cytometry. DMSO served as the control. F) Western blotting was performed to evaluate apoptosis‐related proteins, including Bcl‐2, cleaved Caspase‐3, cleaved Caspase‐9, and Cytochrome C (in both the cytoplasm and mitochondria) in GBM‐3 and T98G temozolomide‐resistant cell lines after treatment with the indicated concentrations of SL for two days. DMSO was used as a control. G) Schematic diagram illustrating the liposome ethanol injection drug‐loading method. Based on the physicochemical properties of SL, this study chose the ethanol injection method to prepare nanoparticles. Soybean phosphatidylcholine, cholesterol, DSPE‐PEG2k‐Biotin, and DSPE‐PEG2k‐HA were used to encapsulate SL, with SL being loaded within the bilayer. DSPE‐PEG2k‐Biotin and DSPE‐PEG2k‐HA were embedded in the surface of the liposomal carrier to form functionalized lipid nanoparticles. Hyaluronic acid (HA) and biotin can specifically bind to the CD44 receptor and biotin receptor (or streptavidin‐modified targeting molecules) that are highly expressed on the surface of blood–brain barrier endothelial cells and glioblastoma cells, respectively. This enables targeted penetration of the blood–brain barrier and targeted drug delivery to glioblastoma. The particle size distribution, zeta potential, and polydispersity index of the liposomal drug carrier were determined using a dynamic light scattering instrument. The drug release profile of the liposomal drug carrier was evaluated using an enzyme‐linked immunosorbent assay (ELISA) plate reader. Pharmacokinetic analysis following intravenous (i.v.) injection in mice was conducted using liquid chromatography‐mass spectrometry (LC‐MS). () In vivo imaging was utilized to examine the enrichment and retention time of liposome‐encapsulated drugs administered via different methods in the mouse head. I) Biotin concentration assays were conducted to measure changes in drug concentration in mouse cerebrospinal fluid over time with various administration methods. J) In vivo imaging was performed to evaluate the therapeutic effects of the SL and TMZ combination in mice and the enrichment of liposome‐encapsulated SL in the mouse brain. The color scale bar on the right represents the signal intensity. K) Overlap analysis of mouse tumor lesions and drug enrichment regions. L) Immunofluorescence analysis was carried out to detect drug carrier enrichment in inflammatory and tumor models. M) The survival rates of mice were recorded. N) An orthotopic implantation experiment was conducted to assess the in vivo tumorigenic capabilities of LN‐229‐Luciferase cells. Mice were subsequently treated with SL, either alone or in combination with TMZ. Representative images of H&E staining are shown. Scale bar: 2 mm. O) Representative images of immunohistochemical staining of tumor tissues in SL and TMZ‐treated mice are presented, using LC3B, P62, cleaved Caspase‐3, TRIM16, and OPTN antibodies. The data were expressed as mean±SD. Student's t‐test was performed to analyze significance. ^*^ *p* < 0.05, ^**^ *p* < 0.01, ^***^ *p* < 0.001.

Temozolomide, an imidazotetrazine prodrug widely employed as a first‐line treatment for glioblastoma (GBM), exhibited limited clinical efficacy due to poor responsiveness and the development of resistance. In our study, we explored the synergistic potential of combining TMZ with Sanggenol L. Using Jin's formula and q values ≥ 1.15, we confirmed a synergistic interaction over an extended period. Results from MTT assays indicated that the combined treatment with TMZ and SL significantly enhanced inhibition of cell proliferation (**Figure** **6**A). Additionally, LC3B double‐label experiments revealed pronounced autophagic flux in TMZ‐resistant strains, which was notably blocked by SL treatment. Western blot (WB) analysis further corroborated that SL inhibited autophagic flux in TMZ‐resistant cells (Figure 6B–D). Flow cytometry and WB results also demonstrated that SL markedly enhanced apoptosis in TMZ‐resistant strains (Figure 6E,F). To evaluate the in vivo therapeutic effects of combining TMZ and SL treatment, orthotopic glioblastoma implantation assays were conducted. These experiments revealed that mice receiving the combination therapy exhibited significantly smaller tumor volumes than those treated with either agent alone (Figure 6F). Based on the physicochemical properties of SL, this study chose the ethanol injection method to prepare nanoparticles. Soybean phosphatidylcholine, cholesterol, DSPE‐PEG2k‐Biotin, and DSPE‐PEG2k‐HA were used to encapsulate SL, with SL being loaded within the bilayer. DSPE‐PEG2k‐Biotin and DSPE‐PEG2k‐HA were embedded in the surface of the liposomal carrier to form functionalized lipid nanoparticles. IR‐780, a near‐infrared dye, was incorporated into the liposome to facilitate drug tracing. Hyaluronic acid (HA) and biotin can specifically bind to the CD44 receptor and biotin receptor that are highly expressed on the surface of blood–brain barrier endothelial cells and glioblastoma cells, respectively.^[^ ^32^, ^33^, ^34^ ^]^ This enables targeted penetration of the blood–brain barrier and targeted drug delivery to glioblastoma (Figure 6G). Pharmacokinetic analysis following intravenous (i.v.) injection in mice was conducted using liquid chromatography‐mass spectrometry (LC‐MS) (Figure S16A and Table S6, Supporting Information). The drug release profile of the functionalized liposomal drug carrier was evaluated using the dialysis method. (Figure S16B, Supporting Information). The biodistribution of the drug carrier after tail vein injection was monitored using a small‐animal in vivo imaging system (Figure S16C, Supporting Information). The particle size distribution (Figure S16D, Supporting Information), zeta potential (Figure S16E, Supporting Information), and polydispersity index of the liposomal drug carrier were determined using a dynamic light scattering instrument (Table S7, Supporting Information). The results demonstrated that the half‐life of SL is consistent with the peak release from the nanoparticles and the peak accumulation in the brain. SL exhibited acceptable metabolic stability with a metabolic half‐life of 3.9 h. Additionally, kidney and liver functions remained unaffected, suggesting minimal systemic toxicity of SL. Through carotid perfusion, tail vein injection, and intraperitoneal injection in C57 mice, we assessed drug distribution. Live imaging and biotin concentration measurements revealed that tail vein injection provided the most consistent brain drug retention and concentration (Figure 6H,I). Importantly, continuous intravenous administration of SL did not cause any notable histopathological alteration in major organs (Figure S17, Supporting Information). An in situ tumor (LN‐229‐Luciferase cell) model was then established in mice, followed by treatment with TMZ, SL, or their combination. Live imaging demonstrated substantial accumulation of SL at the lesion site, and the combination therapy group exhibited significantly reduced tumor growth compared to either monotherapy. (Figure 6J,K; Figure S18, Supporting Information). Immunofluorescence experiments confirmed that preferential accumulation of liposomal SL at tumor sites, with significantly higher localization than adjacent tissue in both inflammatory and tumor models (Figure 6L). Combination therapy of TMZ and SL significantly prolonged the survival of treated mice. H&E staining revealed a notable reduction in tumor size (Figure 6M,N). To further validate the therapeutic effect of SL combined with TMZ, we established a human‐derived glioma xenograft model in nude mice, in which the combination treatment also demonstrated a significant advantage (Figure S19, Supporting Information). while immunohistochemical analysis validated that the protein expression patterns in tumor sections following combination treatment were consistent with in vitro observation. (Figure 6O). In summary, our research highlights the therapeutic potential of SL in combination with TMZ as a promising strategy to tackle TMZ resistance and enhance treatment efficacy in glioblastoma.

## 3 Discussion

Glioblastoma (GBM) is the most aggressive adult brain tumor, marked by high recurrence and resistance to temozolomide.^[^ ^35^, ^36^ ^]^ Although current therapies have extended survival to some extent, the prognosis remains dismal, largely due to TMZ resistance driven by multiple mechanisms, including EGFR overexpression, p53 mutations, and enhanced autophagy.^[^ ^37^ ^]^ Our analysis of 44 clinical glioma samples revealed that LC3B and ATG5 expression increased with tumor grade, while cleaved caspase‐3 decreased, supporting the notion that protective autophagy plays a key role in GBM progression and treatment failure.

Through compound screening, we identified Sanggenol L, a natural flavonoid from mulberry bark, as a selective inhibitor of glioblastoma cell proliferation. Notably, SL not only blocked autophagic flux, confirmed by LC3B double‐labeling, but also induced significant apoptosis, as evidenced by TUNEL staining and caspase activation. These dual actions suggest that SL disrupts the survival advantage conferred by autophagy in TMZ‐resistant GBM cells.

Mechanistic studies revealed that SL targets the TRIM16–OPTN axis, a novel regulatory pathway in glioma biology. SL treatment led to the ubiquitination‐mediated degradation of OPTN, a key receptor in autophagosome–lysosome fusion.^[^ ^38^, ^39^ ^]^ We identified TRIM16 as the E3 ligase responsible for this effect,^[^ ^40^, ^41^ ^]^ and its interference reversed SL‐induced OPTN degradation and autophagy arrest, demonstrating that this axis is essential for SL‐mediated cytotoxicity.

Importantly, SL synergized with TMZ to overcome resistance in GBM‐3 and T98G cells. This combinatorial effect was attributed to enhanced autophagy inhibition and increased apoptosis. To further support its translational potential, we developed a drug delivery formulation using the ethanol injection method, improving SL's solubility and brain penetration. In orthotopic mouse models, SL in combination with TMZ significantly delayed tumor growth and enhanced therapeutic efficacy.

This study is the first to identify TRIM16 as an upstream regulator of OPTN in glioma and to demonstrate that targeting this pathway can reverse TMZ resistance. Notably, when compared to other autophagy inhibitors such as bafilomycin A1—a well‐characterized inhibitor that blocks autophagosome‐lysosome fusion by inhibiting vacuolar‐type H+‐ATPase (V‐ATPase)—SL exhibits distinct advantages. Bafilomycin A1 acts broadly on lysosomal function, which can lead to off‐target effects and systemic toxicity due to the disruption of essential lysosomal processes in normal cells. In contrast, SL specifically targets the TRIM16‐OPTN axis, a pathway more selectively involved in autophagic flux regulation in GBM cells, potentially reducing adverse effects on normal tissues. Furthermore, while bafilomycin A1 primarily inhibits late‐stage autophagy, SL not only blocks autophagic flux but also concurrently induces apoptosis, a dual mechanism that may provide superior efficacy in overcoming TMZ resistance compared to autophagy inhibitors with a single mode of action. This specificity and multi‐targeted activity make SL a more promising candidate for clinical translation in GBM therapy. SL not only acts on a critical node of autophagy regulation but also offers a practical route for drug delivery across the blood–brain barrier, addressing two major challenges in GBM therapy.

In conclusion, our findings reveal a novel molecular mechanism by which SL disrupts protective autophagy and triggers apoptosis in drug‐resistant glioblastoma. By identifying the TRIM16–OPTN axis as a therapeutic target and validating the combination of SL with TMZ, this study provides a new conceptual framework and a feasible therapeutic strategy for overcoming chemoresistance in GBM. Further clinical investigations are warranted to evaluate its translational value.

Although this study confirms that small‐molecule drugs targeting protective autophagy can significantly enhance the killing effect of TMZ on glioblastoma, clinical tumor treatment has entered the era of multimodal combination.^[^ ^42^ ^]^ Targeted intervention of a single pathway is difficult to completely overcome tumor heterogeneity and adaptive resistance. Based on the current research results, we believe that the synergistic application of small‐molecule drug‐TMZ combination regimens and immune checkpoint inhibitors will be a highly promising direction for exploration.

As a typical “cold tumor”, the immune microenvironment of glioblastoma is replete with inhibitory cytokines, numerous immunosuppressive cells, and insufficient effective T cells.^[^ ^43^ ^]^ Moreover, tumor cells often evade immune surveillance by highly expressing molecules such as PD‐L1 and IDO.^[^ ^44^, ^45^ ^]^ Existing studies have shown that while exerting cytotoxic effects, TMZ can induce immunogenic cell death of tumor cells to release tumor‐associated antigens (such as ATP and HMGB1),^[^ ^46^ ^]^ creating conditions for the efficacy of immune checkpoint inhibitors. The small‐molecule drug in this study, by blocking autophagic flux, can not only enhance the chemosensitivity of TMZ, but also may reduce the degradation of intracellular antigens by tumor cells through autophagy inhibition, thereby improving tumor immunogenicity. In addition, autophagy deficiency can inhibit the polarization of macrophages to M2 type and reduce the infiltration of Treg cells, thereby relieving the immunosuppressive state.^[^ ^47^, ^48^ ^]^

Future research can be carried out from three aspects: first, verify the synergistic effect of the three‐drug combination in animal models, focusing on evaluating the dynamic changes in the infiltration pattern of immune cells in the tumor microenvironment (such as the proportion of CD8⁺T cells and cytokine profiles); second, use single‐cell sequencing technology to analyze the selective clearance effect of the combined therapy on tumor cell subsets and clarify the molecular characteristics of the benefited population; finally, explore nano‐delivery systems to achieve targeted co‐delivery of small‐molecule drugs, TMZ, and immune checkpoint inhibitors, to increase the intracranial drug concentration while reducing systemic toxicity. This interdisciplinary research direction may not only break through the bottleneck in the treatment of glioblastoma, but also provide a theoretical basis for the multi‐dimensional combination of “chemotherapy‐autophagy regulation‐immunotherapy”.

## 4 Experimental Section

### Regents and Antibodies

Sanggenol L (DS0100) was procured from LEMEITIAN MEDICINE (Chengdu, China) and dissolved in DMSO. Temozolomide (Cat#HY‐17364) was sourced from MedChemExpress (New Jersey, USA). The following antibodies were purchased from Proteintech (Wuhan, China): anti‐ATG5 (Cat#10181‐2‐AP), anti‐SQSTM1 (Cat#18420‐1‐AP), anti‐CyclinE1 (Cat#11554‐1‐AP), anti‐TRIM16 (Cat#24403‐1‐AP), anti‐Tubulin (Cat#11224‐1‐AP), anti‐HA (Cat#51064‐2‐AP), and anti‐OPTN (Cat#10837‐1‐AP). Additionally, the following antibodies were obtained from Cell Signaling Technology (Boston, MA, USA): anti‐Bcl2 (Cat#32124), anti‐Cleaved Caspase3 (Cat#9664T), Cleaved Caspase‐9 (Cat#20750), anti‐LC3B (Cat#3868T), and anti‐Flag (Cat#8146T). MTT (Cat#M5655), CHX (Cat#C7698), MG132 (Cat#M7449), and DMSO (Cat#D5879) were acquired from Sigma‐Aldrich (St. Louis, MO, USA). The following reagents and kits were purchased from Beyotime (Shanghai, China): DAPI (Cat#C1002), the One Step TUNEL Apoptosis Assay Kit (Cat#C1089), the Hematoxylin and Eosin Staining Kit (Cat#C0105S), the BCA Protein Assay Kit (Cat#P0012), the Cell Lysis Buffer for Western and IP (Cat#P0013), the Crystal Violet Staining Solution (Cat#C0121), HRP goat anti‐mouse antibody (Cat#A0126), HRP goat anti‐rabbit antibody (Cat#A0208), and Alexa Fluor 488‐labeled Goat Anti‐Rabbit IgG (Cat#A0423). Finally, the transfection reagent Lipofectamine 2000 was obtained from Thermo Fisher Scientific (New York, USA).

### Cell Culture

Established GBM cell lines (LN‐229 and T98G), normal astroglial cells (SVGP12), and human embryonic kidney cells (HEK293FT) were obtained from the American Type Culture Collection (ATCC, USA). The patient‐derived GBM cell line GBM‐3 was provided by Dr. Hao W (Da Ping Hospital).^[^ ^35^ ^]^ The GBM‐3 and T98G TMZ‐resistant strains of glioblastoma cells were constructed using the stepwise concentration increase method.

Principle: The principle is to gradually increase the concentration of TMZ, allowing the cells to adapt and develop resistance.

Operational steps:

Initial concentration: Expose glioblastoma cells to a low concentration of TMZ (e.g., 5 µg mL^−1^) and culture for 15–20 days.

Concentration increase: After the cells grow to the logarithmic phase, double the concentration of TMZ successively to 10, 20, 40, 80, and 160 µg mL^−1^. Culture for 15–20 days at each concentration gradient.

Resistant strain selection: Ultimately, select the cell strains that can stably grow at higher TMZ concentrations, which are the resistant strains.

All cell lines were cultured as previously described, confirmed to be mycoplasma‐free, and maintained under standardized conditions.

### Cell Proliferation Analysis

Using the MTT experiments, as previously described, cell viability was evaluated28. Cells were sown onto 96‐well plates with three replicates and 1000 cells per well, and they adhered overnight 20 µL of MTT was applied to the cells at the appropriate times for a 2 h incubation. Following the removal of the culture media and incubation with 150 µL of DMSO, the absorbance was determined using a microplate reader and a 560 nm wavelength.

### EDU Staining

EDU staining was employed to assess cell proliferation following the manufacturer's protocol. A total of 2 × 10⁴ cells were seeded into 24‐well plates, cultured overnight, and then allowed to return to their baseline state. The cells were first incubated with 10 mm EDU for 2 h, followed by fixation with 4% PFA for 15 min, permeabilization with 0.3% Triton X‐100 for 10 min, blocking with 5% bovine serum albumin (BSA) for 1 h, and treatment with Click reaction cocktails for 30 min. Finally, the nuclei were stained with DAPI for 30 min at room temperature before being visualized under a microscope.

### Flow Cytometry

Cells were treated with SL or DMSO for 48 h, after which they were digested, centrifuged, and resuspended in PBS buffer. Following this, the cells were fixed in 75% ethanol for a minimum of 24 h in preparation for cell cycle analysis, which was performed using PI and RNase labeling. Subsequently, flow cytometry was employed for cell detection. Each experimental group consisted of three replicates. For apoptosis analysis, cells were labeled with FITC‐Annexin V and PI for 30 min, followed by detection via flow cytometry. The Annexin V‐FITC/PI Apoptosis Detection Kit (Beyotime, Shanghai) was utilized for apoptosis labeling.

### TUNEL Staining

On coverslips, 2 × 10⁴ cells were cultured and subsequently stained using the One‐Step TUNEL Apoptosis Assay Kit (Beyotime, Shanghai). The cells were treated for 48 h with either SL or DMSO, followed by incubation with 4% paraformaldehyde (PFA) for 30 min, 0.3% Triton X‐100 for 5 min, and 5% bovine serum albumin (BSA) for 1 h. Thereafter, the cells were exposed to the TUNEL test solution (50 µL per well) for 60 min in the dark. Finally, an anti‐fluorescence quenching solution was applied to seal the coverslips, and the results were observed under a fluorescence microscope. The emission wavelength for detection was 570 nm (red fluorescence).

### Transfection and Infection

Short hairpin (sh) RNA targeting TRIM16 was cloned into the pLKO.1‐puro plasmid, with the specific sequences provided in Table S1 (Supporting Information). Flag‐tagged OPTN and TRIM16 constructs were procured from Yubio (Wuhan, China). Transfections were performed using the indicated plasmids, which were introduced into HEK293FT cells via Lipofectamine 2000 (Thermo, USA), following the manufacturer's protocol. Viral supernatants and transfected cells were collected 48 h post‐transfection. For infections, GBM cells were treated with viral supernatants in the presence of polybrene (Sigma, USA). Following two rounds of infection, the cells were stably selected and pooled using puromycin (Sigma, USA).

### Western Blot

Cells were harvested using a cell scraper, washed three times with PBS buffer, and then lysed on ice using RIPA cell lysis buffer. Protein concentration was determined with a BCA protein assay kit after combining 60 µg of protein with loading buffer and heating the mixture in a 100 °C water bath for 15 min. The samples were subsequently subjected to SDS‐PAGE followed by transfer onto a PVDF membrane (Millipore, Germany). The membrane was blocked with 5% BSA at room temperature for 2 h, after which it was incubated with both primary and HRP‐linked secondary antibodies. Finally, the membrane was visualized using an ECL detection system (Clinx, Shanghai).

### Label‐Free Analysis

After treating LN‐229 cells with SL or DMSO for two days, the cells were collected and analyzed using label‐free quantitative proteomic analysis conducted by Shanghai Applied Protein Technology Biotechnology Corporation. The analysis included protein extraction, peptide enzymatic hydrolysis, liquid chromatography‐tandem mass spectrometry (LC‐MS/MS) data collection, and database retrieval. Significant differences in protein expression levels were determined by comparing the number of up‐regulated and down‐regulated proteins between groups. Proteins were classified as up‐regulated if their Fold Change (FC) was > 2.0 and as down‐regulated if their FC was < 0.5, with a *p* < 0.05 serving as the standard for statistical significance.

### Ubiquitination Assay and Protein Turnover Assay

The designated plasmids were co‐transfected into HEK293FT cells to perform the ubiquitination assay. The cells were treated with MG132 for 8 h before being harvested and analyzed using Co‐IP and western blot techniques.

The GBM cells were treated with SL or DMSO for the protein turnover assay. Following incubation, the cells were harvested and analyzed via western blot after exposure to 100 µg mL^−1^ CHX for the designated time intervals.

### Quantitative PCR

Cells were lysed using Trizol reagent, followed by the addition of chloroform and gentle mixing to separate the organic phase. RNA was precipitated from the aqueous phase by adding isopropanol. The resulting RNA pellet was washed with 75% ethanol and dissolved in TE buffer. RNA concentration was then measured, and reverse transcription was carried out using 2 µg of RNA and the GoScript reverse transcription system (Promega, USA). Quantitative real‐time PCR (qRT‐PCR) was performed using SYBR qPCR SuperMix Plus, with GAPDH serving as the internal reference. The primers employed in this study are detailed in Table S2 (Supporting Information).

### Immunofluorescence

The 24‐well plates, each containing a round coverslip, were seeded with GBM cells at a density of 2 × 10⁴ cells per well. The cells were cultured for 24 h, fixed with 4% PFA for 15 min, and treated with 0.3% Triton to enhance permeability. To block non‐specific binding, the cells were incubated with 10% BSA before being treated with an anti‐LC3B antibody, followed by an Alexa Fluor 488‐labeled goat anti‐rabbit secondary antibody. Finally, the nuclei were stained with DAPI before microscopic analysis.

### Colony Formation Assay

Using a colony formation assay, the effect of SL on the colony‐forming ability of GBM cells was evaluated. In this experiment, 1000 cells, along with SL, were seeded into each well of a six‐well plate. After a cultivation period of 2 to 3 weeks, the colonies were stained with crystal violet and subsequently quantified using ImageJ software.

### H&E Staining

The tissue samples underwent a sequential process of fixation, dehydration, embedding, and sectioning. Following this, the sections were stained using a hematoxylin and eosin staining kit (Beyotime, Shanghai) in strict accordance with the manufacturer's instructions.

### Animal Studies and Animal Ethics

The experiments were conducted as previously described. Briefly, thirty‐six 4‐week‐old female BALB/c‐Nude mice (obtained from Slike Jingda Laboratory Animal Co., Ltd., Hunan, China; Animal Qualification Number: SCXK‐2019‐0004) were housed in a specific pathogen‐free (SPF) room. LN‐229‐Luciference cells (1 × 10⁵ cells) were slowly and carefully intracranially injected into the brains of each mouse. After ten days, the mice were treated with tail vein injection with either SL or TMZ (30 mg kg^−1^ day^−1^) every other day for a total of 20 days. DMSO injections were administered to the control group. Prior to brain collection, the mice were anesthetized with isoflurane to minimize pain. Randomized and single‐blind strategies were employed for all measurements, and tumor quantity evaluations were carried out as previously described. The animal experiments conducted in this study were approved by the Institutional Animal Care and Use Committee of Southwest University (Ethics Approval Serial Number: IACUC‐20231109‐02) and strictly adhered to the *Guide for the Care and Use of Laboratory Animals* (Ministry of Science and Technology of China, 2006).

### PDX Model Construction–Sample Acquisition and Processing

Sample acquisition: Samples were obtained from the Third Affiliated Hospital of Chongqing Medical University. The study was reviewed and approved by the hospital's Ethics Committee. After the project obtained ethics approval (Approval No.: Research Ethics Review [2024] No.31), fresh tumor tissue (remaining non‐necrotic tumor tissue after surgical resection, with a volume of ≈0.5–1 cm^3^) was collected during the operation. It was placed in a sterile normal saline preservation solution containing penicillin‐streptomycin (100 U mL^−1^) and transported to the laboratory within 30 min at 4 °C. Sample processing: In a biosafety cabinet, the tumor tissue was rinsed 3 times with sterile normal saline to remove residual blood; the tissue was cut into small pieces of 1–2 mm^3^, added with 0.25% trypsin‐EDTA solution, digested at 37 °C for 15 min. After terminating the digestion, it was passed through a 70 µm cell sieve to collect the single‐cell suspension; the cell concentration was adjusted to 1×10⁷ cells mL^−1^ with DMEM/F12 medium containing 10% fetal bovine serum for later use.

Experimental animals: Female BALB/c nude mice (SPF grade, purchased from Hunan Slack Jingda Laboratory Animal Co., Ltd., animal license number: SCXK‐2019‐0004) aged 4–6 weeks were selected. They were raised in an SPF‐grade animal room with a temperature of 22 °C–25 °C, humidity of 50%–60%, a 12 h light‐dark cycle, and free access to food and water. The animal experiment has been approved by the Animal Ethics Committee of Southwest University (ethical approval number: IACUC‐20231109‐02) and strictly follows the Guide for the Care and Use of Laboratory Animals. Tumor cell inoculation: After depilation and disinfection of the right axilla of the nude mice, 0.2 mL of the above tumor single‐cell suspension was aspirated with a 1 mL syringe and injected subcutaneously; Model monitoring: After inoculation, the mental state and weight changes of the nude mice were observed weekly, and the tumor volume was measured with a vernier caliper (volume = length × width^2^ / 2). When the tumor volume reached 100–200 mm^3^, the PDX model was determined to be successfully constructed; after the model was successful, passage (repeating the above inoculation steps with fresh tumor tissue) or subsequent drug sensitivity experiments could be carried out.

### GST‐Pulldown

The plasmids specified were transformed into Rosetta DE3 cells. Isopropyl β‐D‐thiogalactoside was utilized to induce the expression of the designated proteins. Glutathione S‐transferase (GST)‐tagged proteins were pre‐bound to GST‐purified resin, while the lysate containing His‐tagged proteins was gradually passed through the resin bound to the GST‐tagged proteins. After three thorough washes with PBS buffer, the proteins were eluted. A Western blot assay was then performed to confirm whether the proteins interacted with one another.

### Bimolecular Fluorescence Complementation BIFC

The segmented fluorescent proteins, consisting of the N‐fragment and C‐fragment, were fused to the target proteins. The resulting fusion vector was subsequently transferred into HEK293FT cells for expression. Fluorescence was observed 36 h post‐transfection.

### Duo‐Link Pla

Before initiating the procedure, cells were deposited onto slides and subjected to fixation, restoration, and permeability pretreatment. After blocking with Duo‐link blocking buffer, the slides were incubated overnight at 4 °C with the specified antibody mixture. Subsequent steps were carried out in accordance with the manufacturer's protocol.

### Mitochondrial Membrane Potential

A Mitochondrial Membrane Potential Assay Kit utilizing JC‐1 was employed to detect alterations in mitochondrial membrane potential, following the manufacturer's protocol. A total of 2 × 10⁴ cells were seeded into 24‐well plates, incubated overnight, and subsequently returned to their basal state. The cells were then exposed to SL or DMSO treatments for a duration of 48 h. Finally, the results were analyzed using a fluorescence microscope.

### Fluo‐4 AM Calcium Ion Fluorescent Probe Kit Detection

The Fluo‐4 AM Calcium Ion Fluorescent Probe Kit was utilized to assess changes in intracellular calcium concentrations following the manufacturer's protocol. A total of 2 × 10⁴ cells were seeded into 24‐well plates, incubated overnight, and subsequently allowed to return to their baseline state. The cells were then treated with either SL or DMSO for 48 h. Finally, the results were observed using a fluorescence microscope.

### Reactive Oxygen Species Assay Kit Detection

The Reactive Oxygen Species Assay Kit was utilized to detect changes in intracellular reactive oxygen species concentrations, following the manufacturer's guidelines. A total of 2 × 10⁴ cells were seeded into 24‐well plates, cultured overnight, and subsequently allowed to stabilize. The cells were then treated with either SL or DMSO for a duration of 48 h. The results were observed and analyzed using a fluorescence microscope.

### In vitro Protein Ubiquitination Assays

In vitro protein ubiquitination assays were employed to simplify and isolate the complex in vivo environment, enabling direct interaction between the E3 ubiquitin ligase and substrate proteins. The purified E3 ubiquitin ligase and substrate protein were incubated with ubiquitin (UB), E1, E2, ATP, and the buffer provided in the kit, following the kit's protocol. Subsequently, the ubiquitination status of the substrate protein was analyzed.

### Jin's formula

The drug's combined effect in vitro through Jin's formula was assessed. The Jin's formula is as follows:

(1) $$q=\frac{E\left(A+B\right)}{\left(EA+EB-EA*EB\right)}$$

Annotate:

q: Efficiency index, *E*(*A*+*B*): Combined inhibition rate, *EA*: Drug A inhibition rate, *EB*: Drug B inhibition rate (*q*<0.85:antagonism effect, 0.85≤q<1.15:sunperosition effect, q≥1.15:synergistic effect.

### In Vivo Imaging

The in vivo imaging experiment was conducted using the IVIS SpectrumCT system, employing BALB/c nude mice as the animal model. Glioblastoma LN‐229 cells, tagged with luciferase, were injected directly into the brains of the mice, and in vivo imaging was carried out following tumor formation. Before the experiments, the mice underwent a 12 h fasting period and were subsequently anesthetized via intraperitoneal injection of a 0.7% sodium pentobarbital solution. Following anesthesia, D‐luciferin potassium salt was administered as a substrate at a dosage of 150 mg kg^−1^ of body weight. After a 10 min interval, the mice were placed in the imaging dark box, and imaging was performed using designated excitation and emission filters. The imaging parameters included a 1 min exposure time, carefully set to ensure signal stability. Image analysis was conducted using the Living Image software, which was utilized to calculate both the luminescence area and the total photon count.

### Synthesis and Characterization of Functionalized Liposomes

Lipid nanoparticles (LNPs) are one of the most successful carriers to date. In this study, soybean phosphatidylcholine, cholesterol, DSPE‐PEG2k‐Biotin, DSPE‐PEG2k‐HA, and IR‐780 were used to encapsulate SL, with SL being loaded within the bilayer to form functionalized liposomal nanoparticles. Based on the physicochemical properties of SL, the ethanol injection method was chosen to prepare the nanoparticles in this study, with the specific procedures as follows:

1. According to the molar ratio of soybean phosphatidylcholine: cholesterol: DSPE‐PEG2k‐HA: DSPE‐PEG2k‐Biotin of 10:2:1, accurately weigh (1) 10 mg of soybean phosphatidylcholine, 1 mg of cholesterol, 3.6 mg of DSPE‐PEG2k‐HA, 3.6 mg of DSPE‐PEG2k‐Biotin, 2 mg of IR‐780, and 5 mg of SL, and dissolve them in 100 µL of anhydrous ethanol with stirring until uniform.
2. Quickly inject the above mixture into 900 µL of PBS and stir at room temperature for 10 min to form a liposomal colloidal solution.
3. Transfer the prepared liposomal colloidal solution into a dialysis bag and dialyze overnight. Finally, filter through a 0.22 µm filter membrane for later use. Three parallel groups were prepared to determine the encapsulation efficiency (EE) and drug loading (DL).

### Zeta Potential and Dynamic Light Scattering Analysis

The surface charge, hydrodynamic particle size distribution, and polydispersity of the prepared liposomes were tested. At room temperature, the samples were dispersed in ultrapure water, and the sample concentration was adjusted to be clear and transparent. Detection and analysis could only be performed when a clear light path was visible under laser irradiation. Each sample was tested in triplicate.

### Drug Release Profile

Three milliliters of the liposome‐encapsulated drug were divided into multiple centrifugal ultrafiltration tubes, with 200 µL in each tube. The liposome‐encapsulated drug samples were added to the release medium at a drug concentration of 2 mg mL^−1^, with three parallel samples set for each time point. The tubes containing the samples were placed in a constant‐temperature water bath shaker, which was set at 37 °C with a shaking frequency of 80 rpm. At the predetermined time points (0, 5, 15, 30 min, 1, 2, 4, 8, 10, 12, and 24 h), one group of centrifugal ultrafiltration tubes was taken out and immediately centrifuged at 4000 rpm for 3 min to separate the supernatant from the liposomes. One hundred microliters of the supernatant was taken out, and 100 µL of dimethyl sulfoxide (DMSO) was added. After mixing well, the mixture was transferred to a 96‐well plate. The drug standard was dissolved in DMSO to prepare a series of standard solutions with gradient concentrations (0.0008–2 mg mL^−1^). Two hundred microliters of the standard solution was added to the 96‐well plate, with three parallel samples set for each concentration. The absorbance of the standard solutions and samples was measured using a microplate reader. A standard curve was plotted with the concentration of the standard solution on the x‐axis and the absorbance on the *y*‐axis. The drug concentration of each sample at each time point was calculated based on the standard curve equation.

### Pharmacokinetics

C57BL/6J male mice, aged 6–8 weeks, were used. After 3 days of adaptive housing, the mice were randomly assigned to different groups according to their body weights, with the day of grouping designated as Day 0 (D0).

On D0, mice received a single intravenous (i.v.) injection via the tail vein. The drug dose was 3 mL kg^−1^, with an injection volume of 5 mL kg^−1^.

Blood samples were collected from each mouse at 10 time points: 0 h (predose), 5, 15, 30 min, 1, 2, 4, 6, 12, and 24 h. The volume of blood collected at each time point was ≈60 µL. Plasma was separated from the blood samples (yielding about 20–30 µL) for quantitative analysis by liquid chromatography‐mass spectrometry (LC‐MS). LC‐MS was used to determine the concentration of the analyte in the blood at different time points.

### Statistics Analysis

All data were independently presented three times and analyzed using GraphPad Prism 8. The results are expressed as Mean ± SD. Statistical significance was evaluated on independent samples using the Student's unpaired t‐test, with significance thresholds set as follows: *p* < 0.05 (*), *p* < 0.005 (**), and *p* < 0.0005 (***).

## Conflict of Interest

The authors declare no conflict of interest.

## Author Contributions

H.C., J.H., and X.H. contributed equally to this work. H.C., J.H., X.H., and N.H. contributed to conceptualization. H.C., J.H., X.H., N.H., and Z.X. contributed to Methodology. H.C., J.H., and N.H. contributed to investigation. H.C., J.H., X.H., and N.H. visualized the project. H.C., J.H., X.H., N.H., Y.D., M.X., J.X., and Y.W. contributed to supervision. H.C., J.H., X.H., and M.X. wrote the original draft. HC and YZ wrote, reviewed & edited the final manuscript.

## Supporting information



Supporting Information

## Data Availability

The data that support the findings of this study are available from the corresponding author upon reasonable request.
