# Supplementary material for: Sanggenol L Enhances Temozolomide Drug Sensitivity by Inhibiting Mitophagy and Inducing Apoptosis Through the Regulation of the TRIM16‐OPTN Axis in Glioblastoma
Source: Adv Sci (Weinh). 2025 Sep 24;12(45):e02915. doi: 10.1002/advs.202502915 (PMC12677614; doi:10.1002/advs.202502915)
Supplement: Supplementary file 1 — Supporting Information [file ADVS-12-e02915-s001.docx]

Supporting Information for

**Sanggenol L Enhances Temozolomide Drug Sensitivity by Inhibiting Mitophagy and Inducing Apoptosis through the Regulation of the TRIM16-OPTN Axis in Glioblastoma**

Hongbo Chang *et al.*

*Corresponding author. Email: hcui@swu.edu.cn

**
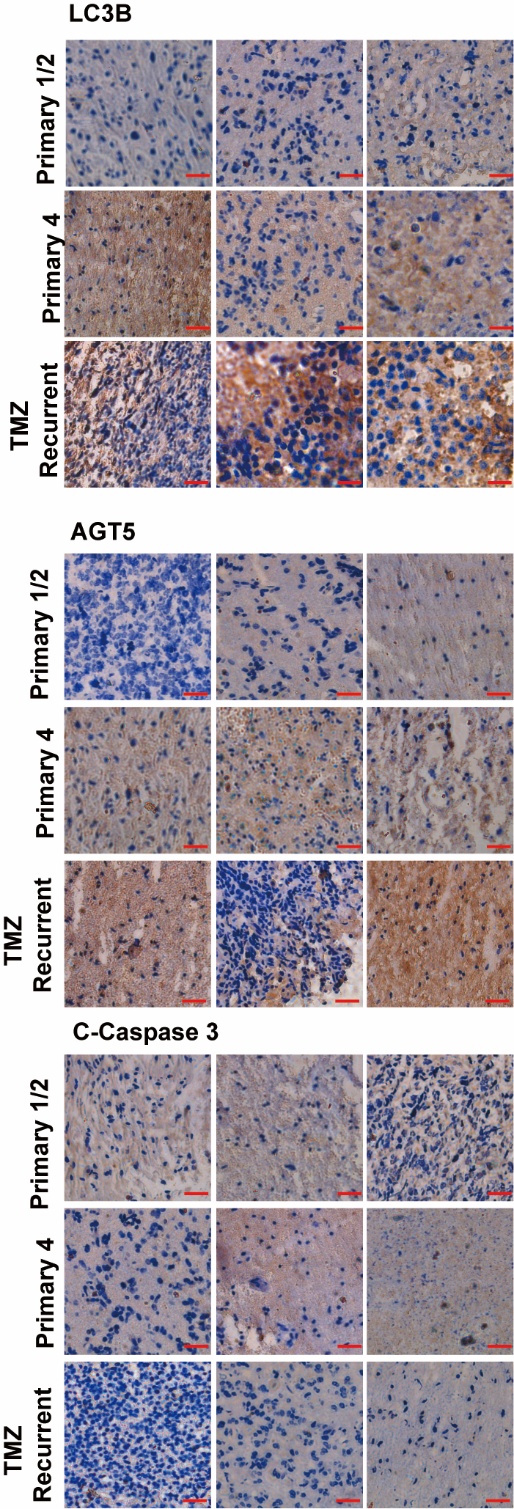
**

**Fig. S1.**

Representative images showcasing immunohistochemical staining of glioma clinical specimens using antibodies against LC3B, AGT5, and C-Caspase-3. Scale bar: 100 μm


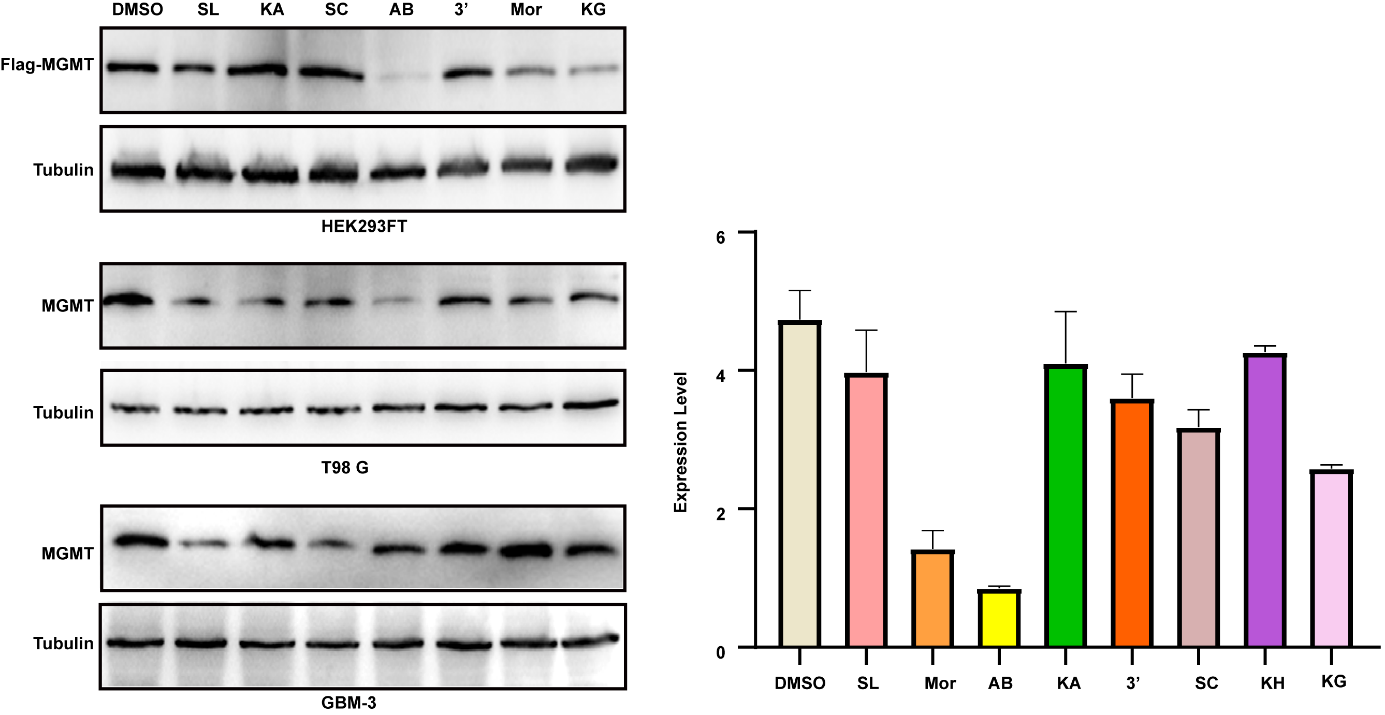


**Fig. S2.**

Western blotting was performed to assess changes in MGMT expression levels in MGMT-overexpressing HEK293FT, T98G, and GBM-3 cell lines following drug treatment. Fluorescence intensity in MGMT-overexpressing HEK293FT cells after drug treatment was measured by fluorescence signal detection. （Sanggenol L: SL; Morusinol: Mor; Albanol b: AB; Kuwanon A: KA; PSX003: 3’; Sanggenon C: SC; Kuwanon H: KH; kuwanon G: KG）

**
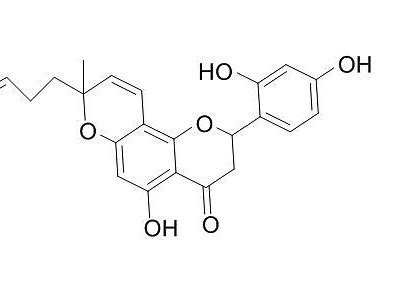
**

**Fig. S3.**

The chemical structure of SL.

**
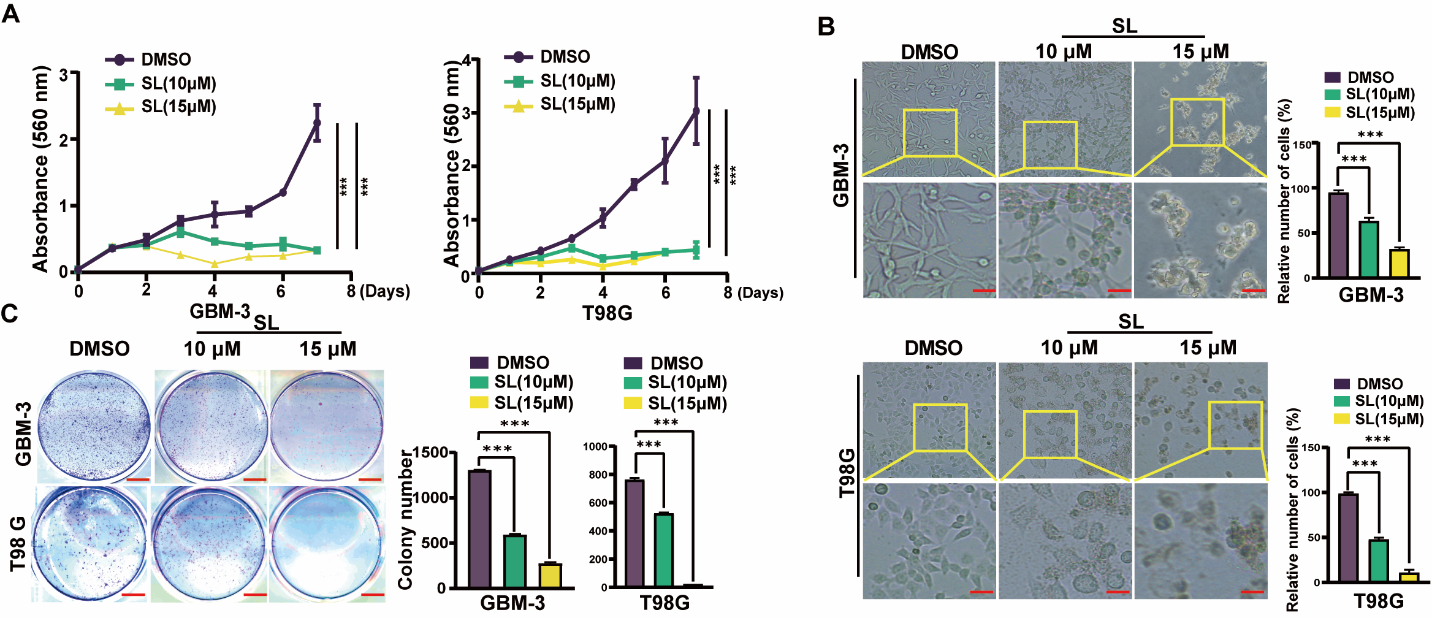
Fig. S4.**

(**A**) Viability of GBM-3 and T98G cells following treatment with 10 μM and 15 μM SL. DMSO was used as the control. (**B**) Cellular morphology of GBM-3 and T98G cells after 48 hours of incubation with the specified concentrations of SL or DMSO. Scale bars = 10 μm. DMSO served as the control. (**C**) Impact of the specified concentrations of SL or DMSO on in vitro colony formation in GBM-3 and T98G cells. SL promotes apoptosis in GBM cells.

**
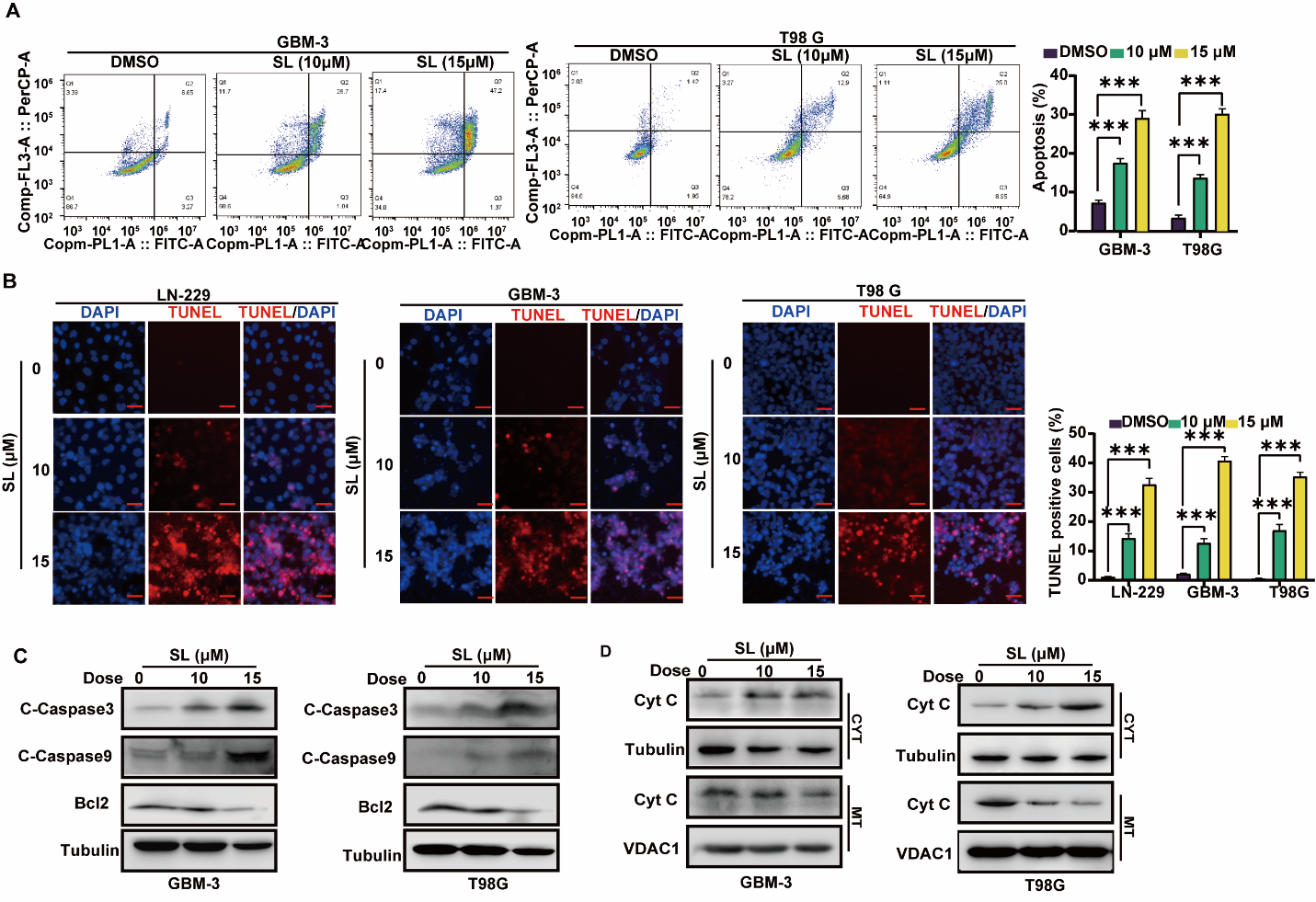
Fig. S5.**

(**A**) The apoptosis of GBM-3 and T98G cells treated with SL (10 μM and 15 μM) or DMSO for two days was analyzed using flow cytometry, with DMSO serving as the control. (**B**) A representative TUNEL staining image of GBM-3 and T98G cells following treatment with SL (10 μM and 15 μM) or DMSO for two days. Scale bars = 50 μm. DMSO was used as the control. (**C, D**) Western blot assays were conducted to evaluate apoptosis-related proteins, including Bcl-2, cleaved Caspase-3, cleaved Caspase-9, and Cytochrome C (in both the cytoplasmic and mitochondrial fractions), in GBM-3 and T98G cells after treatment with the specified concentrations of SL for two days. DMSO served as the control.

**Fig. S6.**

(**A**) Immunofluorescence staining of LC3B (green) was conducted in GBM-3 and T98G cells treated with or without SL (10 μM) for 2 days. The cell nuclei were counterstained with DAPI (blue). Scale bars = 10 μm. (**B**) Immunofluorescence analysis was performed on GBM-3 and T98G cells transiently transfected with mRFP-EGFP-LC3, followed by treatment with DMSO, Rapa (1 nM), CQ (10 μM), KuH (30 μM), or a combination of Rapa (1 nM) and SL (10 μM). Scale bar = 20 μm. Cells treated with rapamycin and CQ served as positive and negative controls, respectively. (**C**) Western blot assays were carried out to assess autophagy-related proteins, including SQSTM1, AGT5, and LC3B, in GBM-3 and T98G cells after exposure to the indicated concentrations of SL for specified durations over 2 days. DMSO was used as a control. (**D**) Expression of LC3B in GBM-3 and T98G cells following treatment with SL and bafilomycin A1 (Baf A), detected via Western blot (WB).


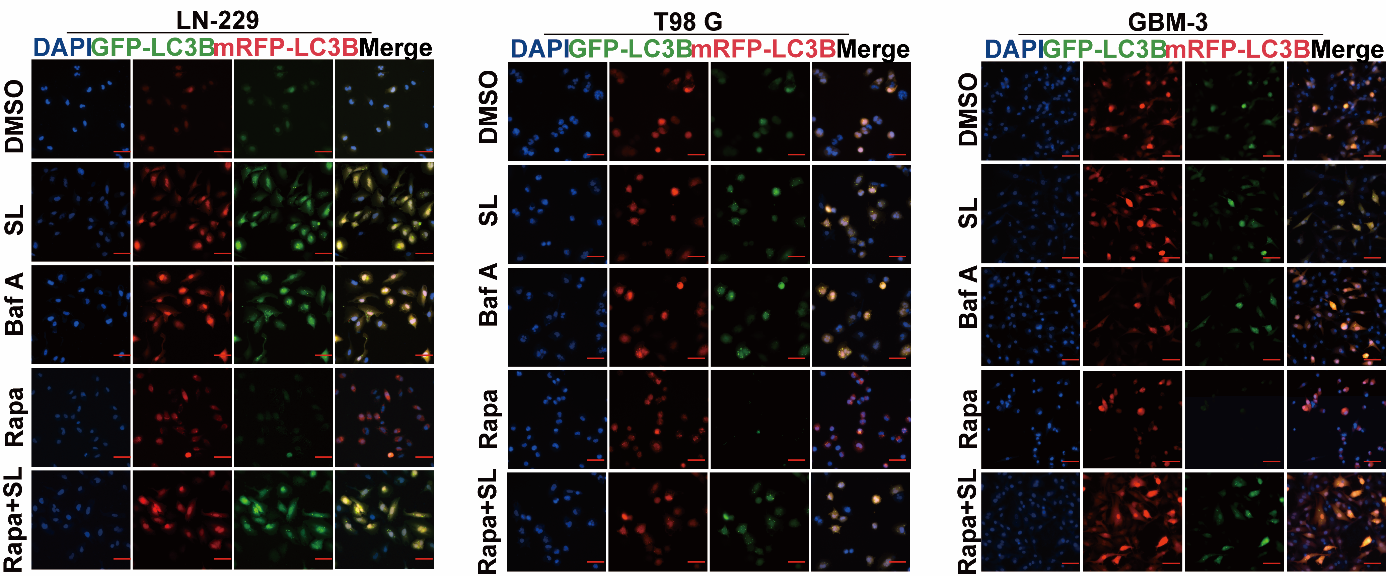


**Fig. S7.**

Immunofluorescence analysis of LN-229, T98 G, and GBM-3 cells transiently transfected with mRFP-EGFP-LC3, followed by treatment with DMSO, rapamycin (Rapa, 1 nM), chloroquine (CQ, 10 μM), SL (10 μM), or a combination of Rapa (1 nM) and SL (10 μM). Scale bars = 100 μm. Cells treated with rapamycin and CQ were used as positive and negative controls, respectively.

**
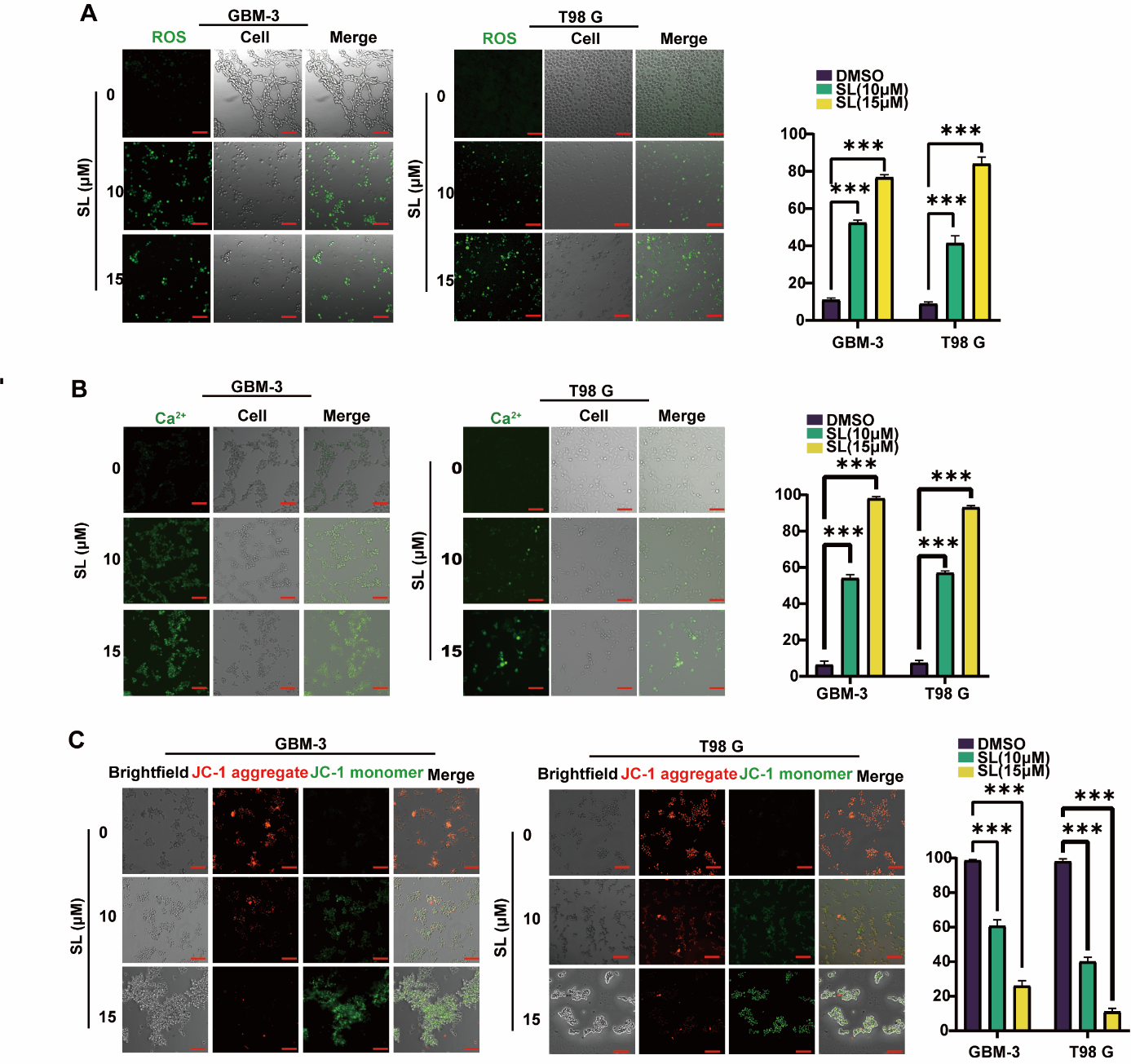
Fig. S8.**

(**A**) Fluorescence imaging and statistical analysis of cells stained with a Reactive Oxygen Species (ROS) assay following treatment with SL at concentrations of 10 μM and 15 μM. DMSO served as the control. (**B**) Fluorescence imaging and statistical analysis of cells stained with the Fluo-4 AM calcium ion fluorescent probe after treatment with SL at concentrations of 10 μM and 15 μM. DMSO served as the control. (**C**) Fluorescence imaging and statistical analysis of cells stained with JC-1 following treatment with SL at concentrations of 10 μM and 15 μM. DMSO served as the control. (Red: mitochondrial aggregates; green: mitochondrial monomers). Scale bar: 50 μm.


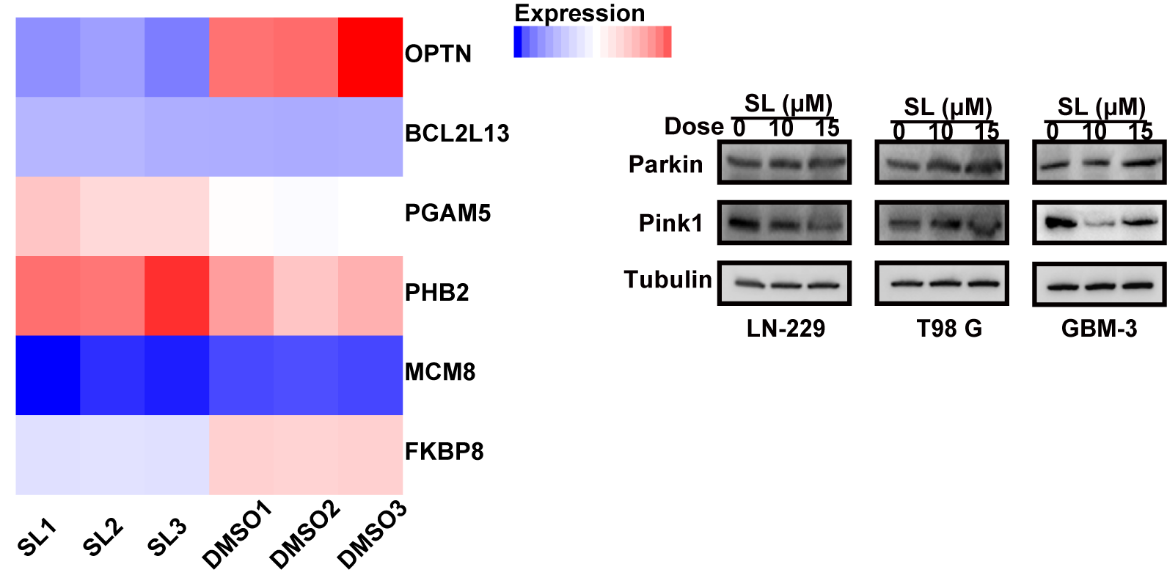


**Fig. S9.**

Proteomic analysis of glioblastoma samples treated with SL yielded a heatmap showing the expression of autophagy-related receptor proteins. Western blotting was used to measure Parkin and Pink1 protein levels in LN-229, T98G, and GBM-3 cell lines.


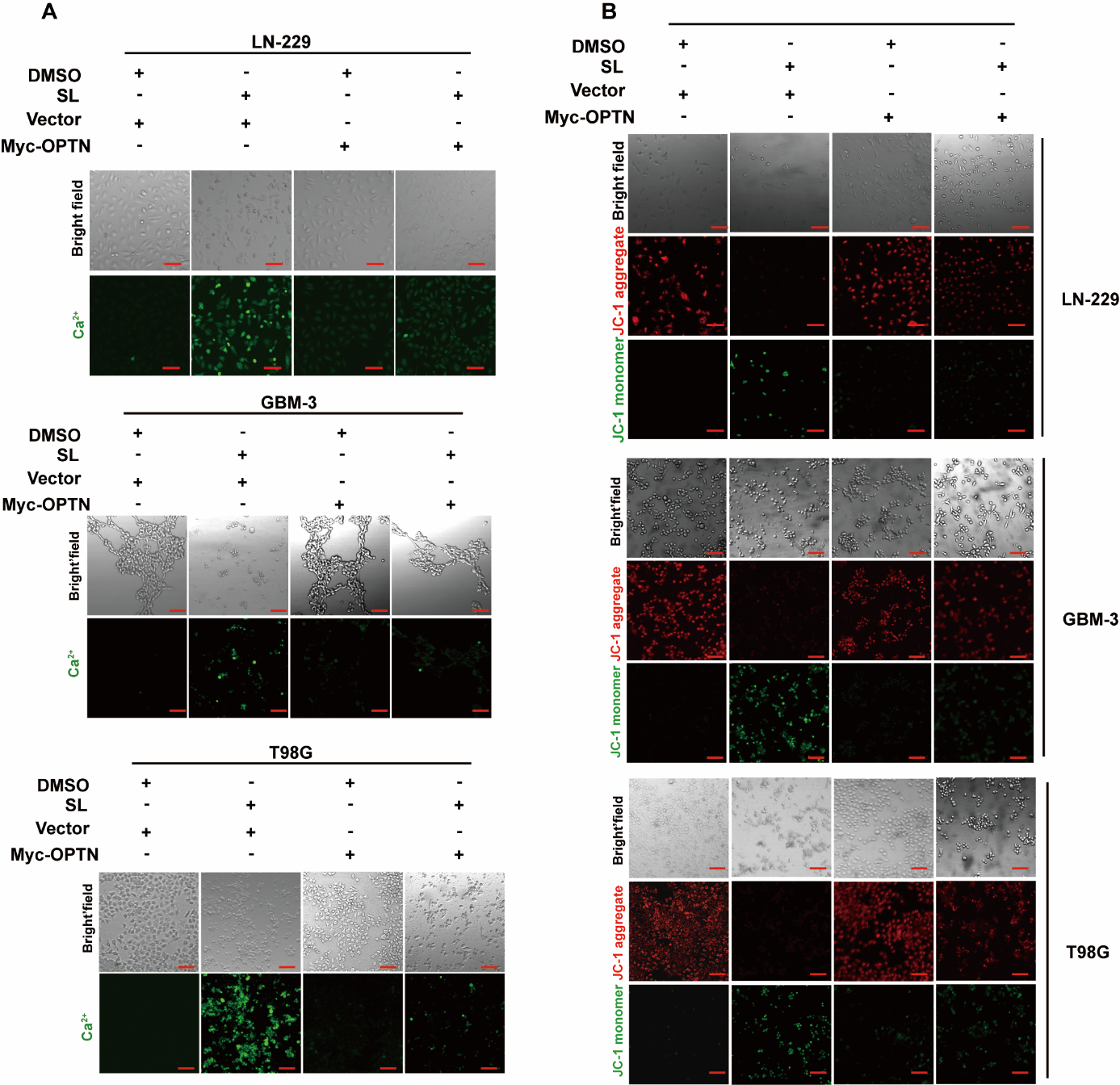


**Fig. S10.**

(**A**) Fluorescence imaging and statistical analysis were performed on cells stained with the Fluo-4 AM calcium ion fluorescent probe following treatment with SL (10 μM and 15 μM), using DMSO as the control. (**B**) Fluorescence imaging and statistical analysis were conducted on cells stained with JC-1 after treatment with SL (10 μM and 15 μM), with DMSO serving as the control. (Red indicates mitochondrial aggregates; green indicates mitochondrial monomers.) Scale bar: 50 μm.


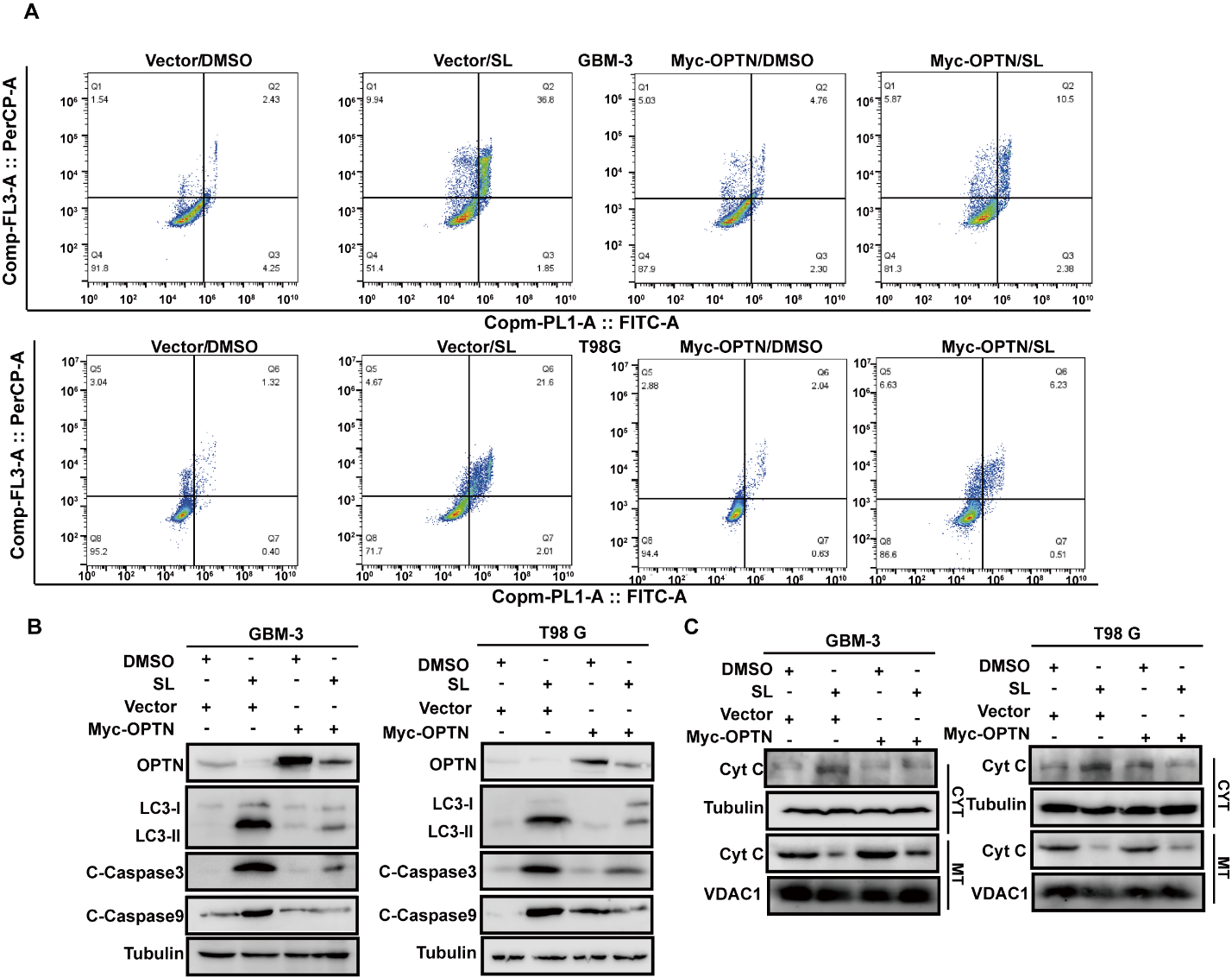


**Fig. S11.**

(**A**) The apoptosis of GBM-3 and T98G cells treated with SL (10 μM and 15 μM) or DMSO for two days was analyzed using flow cytometry, with DMSO serving as the control. (**B, C**) Western blot assays were conducted to assess apoptosis-related proteins, including Bcl-2, Cleaved Caspase-3, Cleaved Caspase-9, and Cytochrome C (both in the cytoplasm and mitochondria), in GBM-3 and T98G cells following treatment with the specified concentrations of SL for two days. DMSO was used as the control.


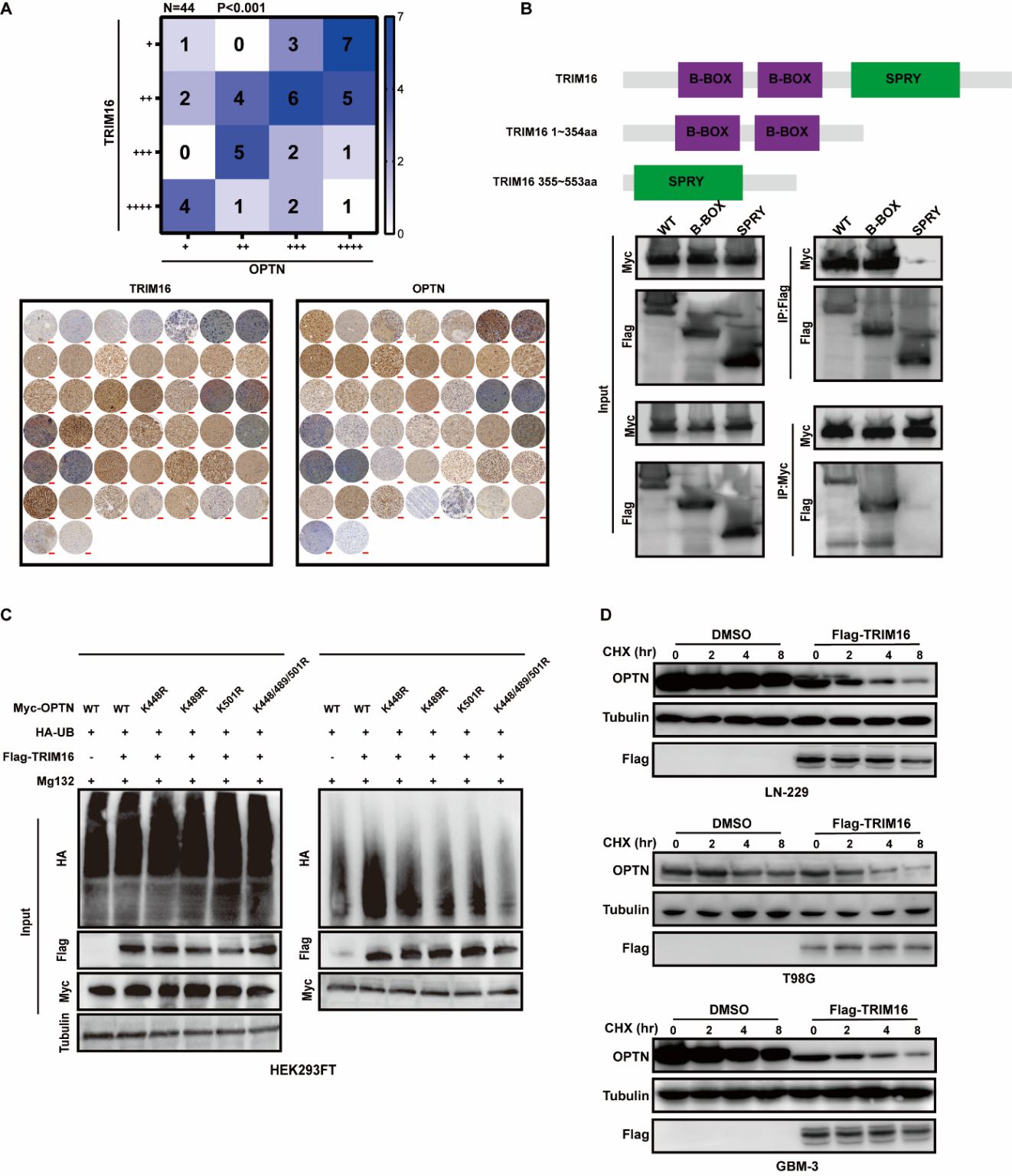


**Fig. S12.**

(**A**) Representative immunohistochemical staining images of glioma clinical specimens were obtained using TRIM16 and OPTN antibodies. Scale bar: 100 μm (**B**) Truncation and domain deletion of OPTN and TRIM16 vectors were utilized to identify the interaction domain. (**C**) GBM-3 and T98G cells were treated with SL (10 μM) or DMSO, followed by treatment with CHX (100 μg/mL) for the specified time intervals. The cells were then harvested, and the OPTN turnover rate was analyzed through western blot analysis. Grayscale values were annotated on the WB bands, with 0 h serving as the baseline for each group. DMSO was used as the control.


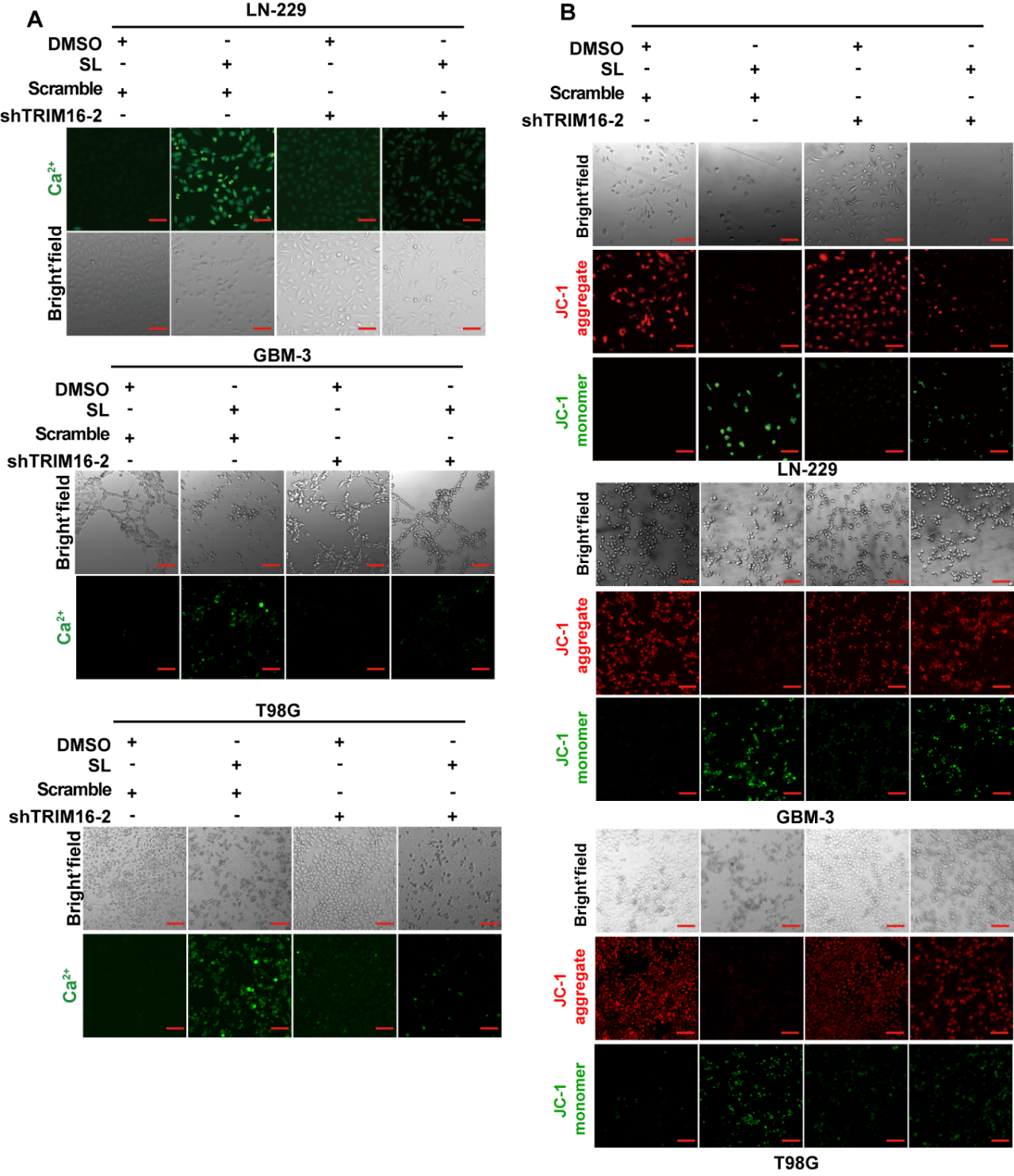


**Fig. S13.**

(**A**) Fluorescence imaging and statistical analysis of cells stained with the Fluo-4 AM calcium ion fluorescent probe following treatment with SL (10 μM and 15 μM). DMSO was used as the control. (**B**) Fluorescence imaging and statistical analysis of cells stained with JC-1 following treatment with SL (10 μM and 15 μM). DMSO was used as the control. (Red: mitochondrial aggregates; green: mitochondrial monomers). Scale bar: 50 μm.

**
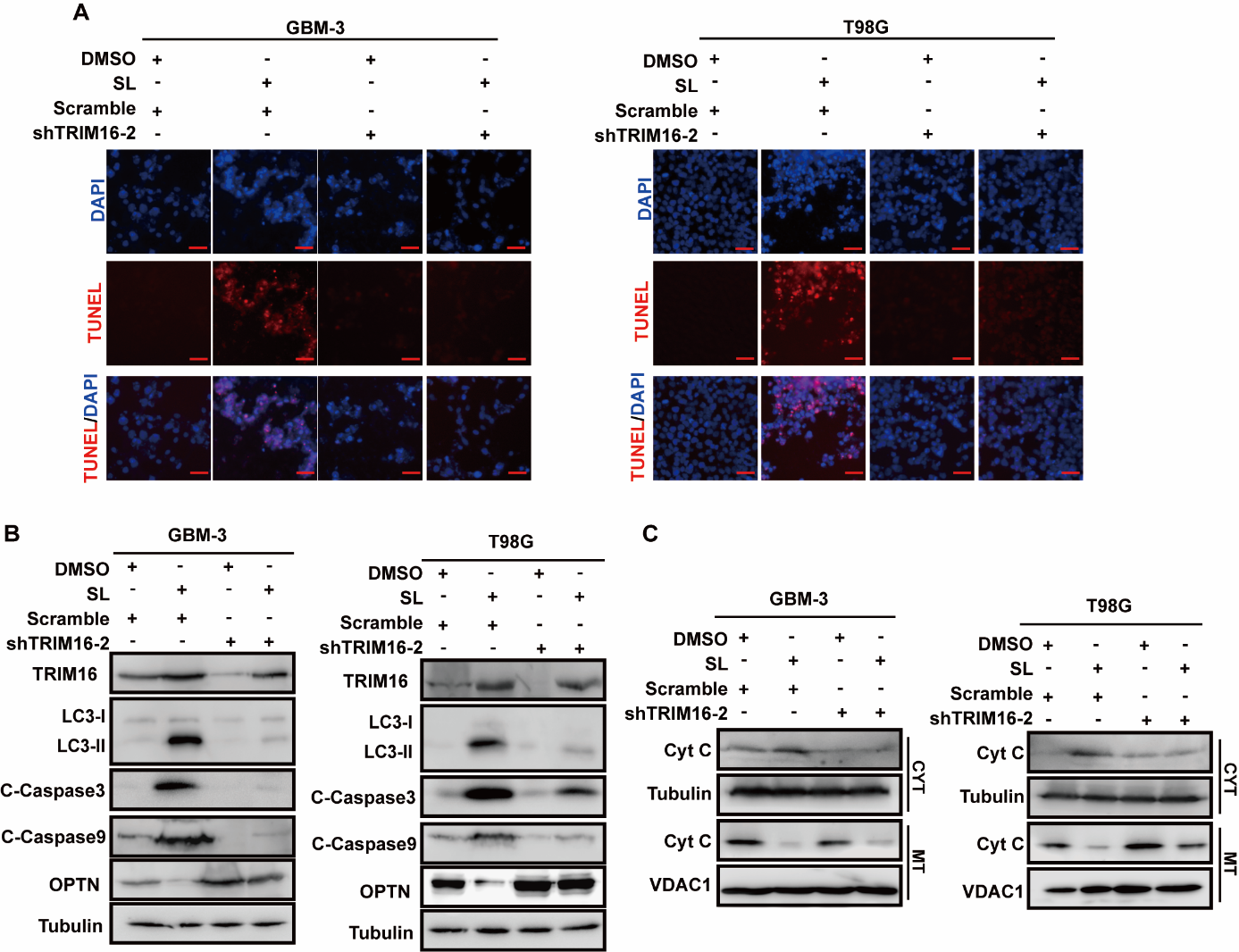
Fig. S14.**

(**A**) The apoptosis of GBM-3 and T98G cells, treated with SL at concentrations of 10 μM and 15 μM or with DMSO for two days, was assessed using TUNEL staining, with DMSO serving as the control. Scale bar: 50 μm (**B, C**) Western blot analyses were conducted to evaluate apoptosis-related proteins, including Bcl-2, cleaved Caspase-3 (C-Caspase3), cleaved Caspase-9 (C-Caspase9), and Cytochrome C (in both the cytoplasm and mitochondria), in GBM-3 and T98G cells following treatment with the specified concentrations and durations of SL for two days. DMSO was used as the control.

**
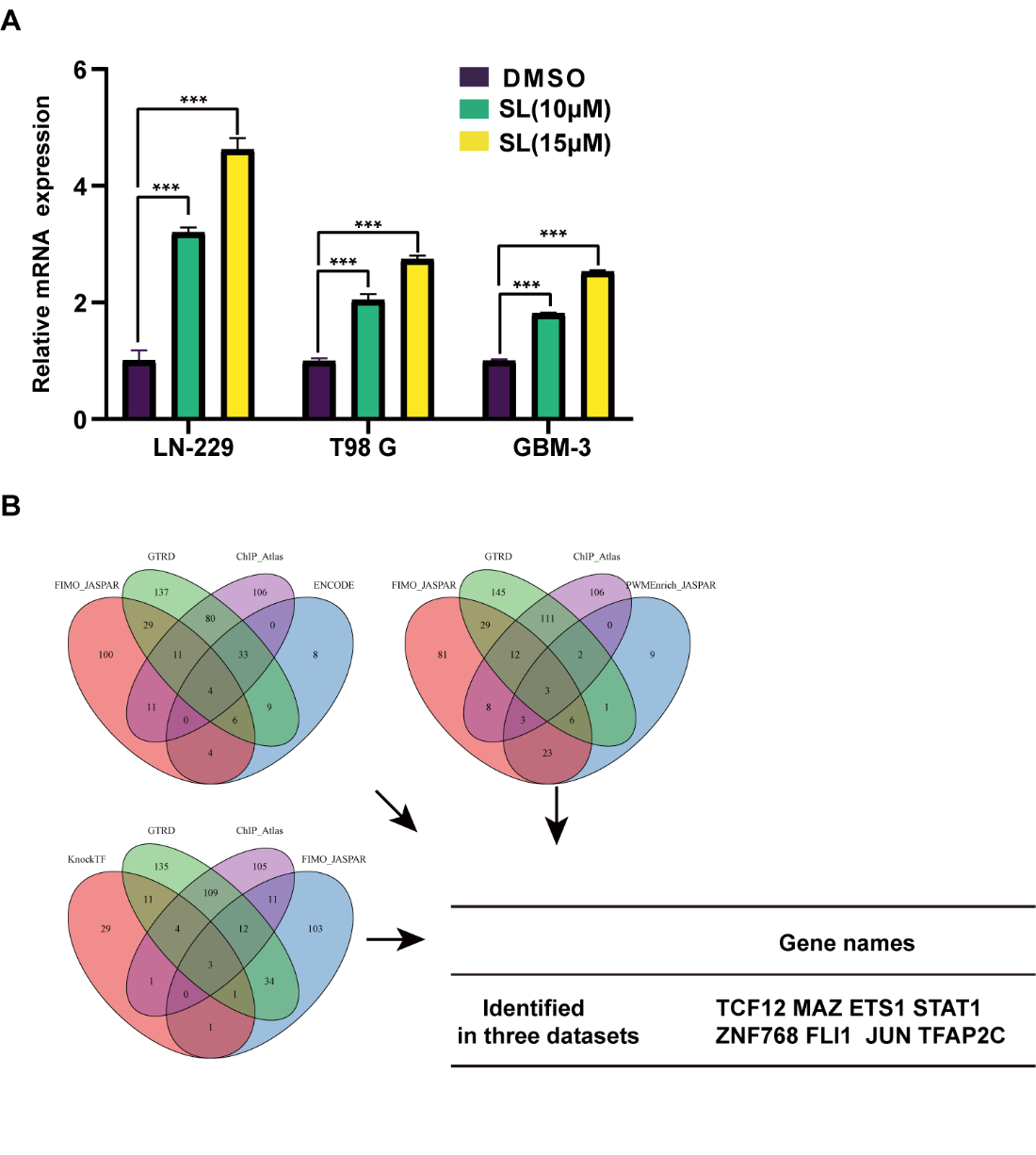
**

**Fig. S15.**

(**A**) Quantitative real-time PCR analysis of relative mRNA expression in glioblastoma cell lines LN-229, T98G, and GBM-3 after treatment with DMSO (control), SL (10 μM), or SL (15 μM). Data are presented as mean ± SD, and statistical significance was determined by Student’s t-test (***p < 0.001). (**B**) Venn diagrams showing the overlap of transcription factors predicted or identified from different datasets (GTRD, ChIP_Atlas, ENCODE, FIMO_JASPAR, WAMEnrich_JASPAR, KnockTF). The intersection of three datasets yielded eight common transcription factors: TCF12, MAZ, ETS1, STAT1, ZNF768, FLI1, JUN, and TFAP2C.


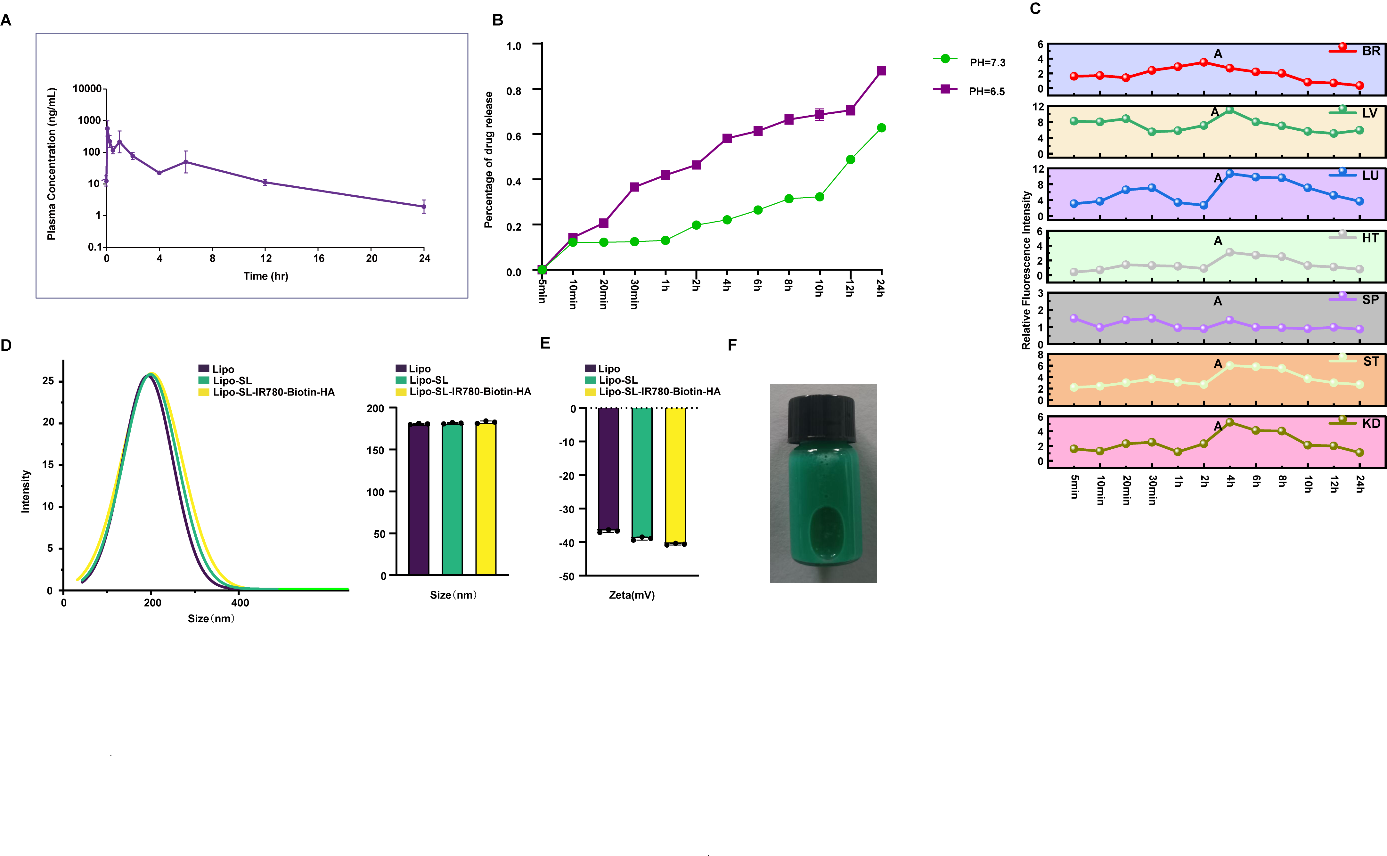


**Fig. S16.**

(**A**) Pharmacokinetic analysis of drug concentration in nude mouse plasma over time post-injection. (**B**) Drug-release profile of functionalized liposomes. (**C**) Organ distribution of functionalized liposomes following tail-vein injection in nude mice. (**D-F**) Average particle size and surface charge of functionalized liposomes.

**
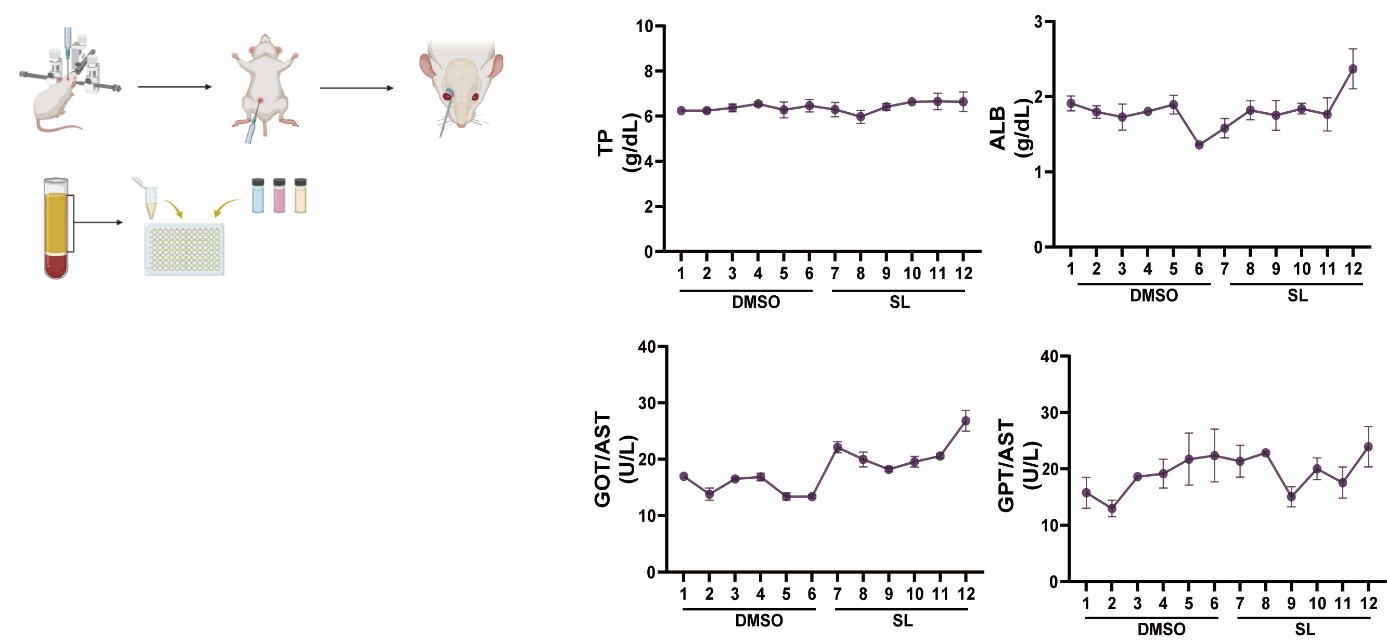
Fig. S17.**

After intracerebral tumor implantation in mice, the SL drug carrier was delivered via tail vein injection. Blood was then collected through ocular sampling to evaluate relevant indicators.


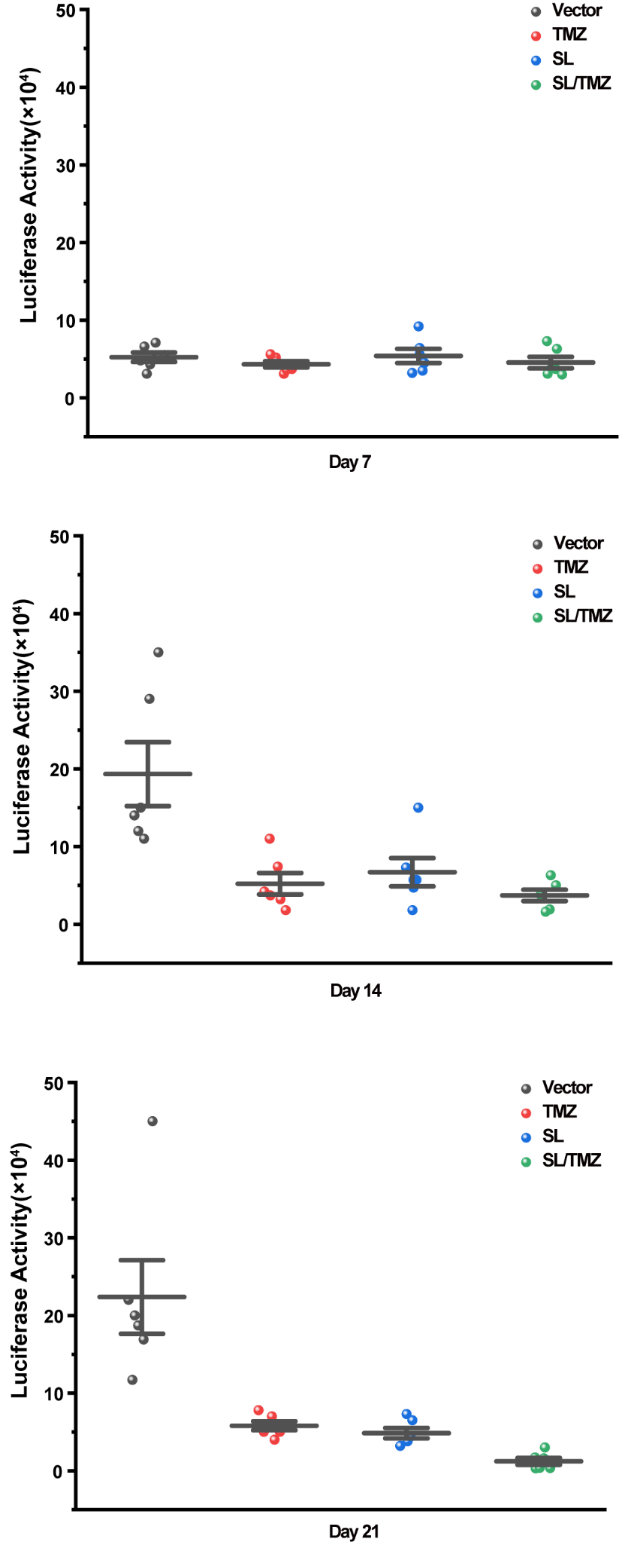


**Fig. S18.**

Quantitative analysis of orthotopic tumor formation in nude mice using in vivo imaging.


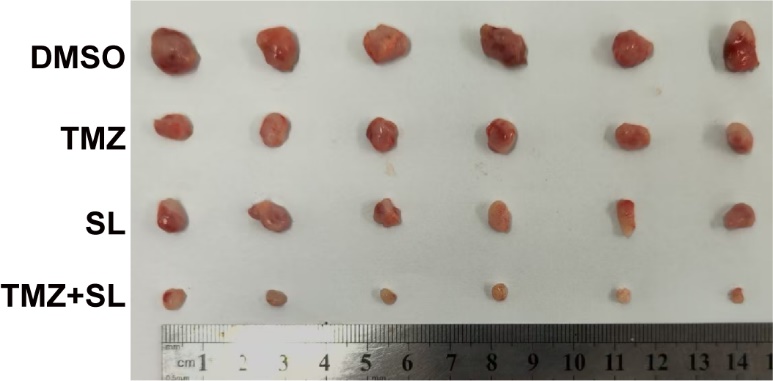


**Fig. S19.**

Patient-derived glioblastoma tissue was subcutaneously implanted into the right flank of mice to establish xenograft tumors. When tumors became palpable, the mice were randomly divided into four groups and treated with SL, TMZ, or their combination, using DMSO as a control. Tumor images are shown.

**Supplementary Table S1.** Small molecule mulberry active substances related to cancer

| Molecule Name | CAS |
| --- | --- |
| Sanggenone C | 80651-76-9 |
| Sanggenol O | 426211-27-0 |
| Kuwanon B | 62949-78-4 |
| Sanggenol A | 174423-30-4 |
| Sanggenol L | 329319-20-2 |
| Sanggenone H | 86450-80-8 |
| (2S)-6-[(1S,5R,6S)-6-(2,4-dihydroxybenzoyl)-5-(2,4-dihydroxyphenyl)-3-(4-methylpent-3-enyl)cyclohex-2-en-1-yl]-2-(2,4-dihydroxyphenyl)-5,7-dihydroxy-2,3-dihydrochromen-4-one | 85698-31-3 |
| Kuwanon K | 88524-66-7 |
| Mulberroside C | 102841-43-0 |
| Mulberroside F | 193483-95-3 |
| Kuwanon A | 62949-77-3 |
| Kuwanon E | 68401-05-8 |
| Kuwanon H | 76472-87-2 |
| Kuwanon T | 100187-66-4 |
| Morusinol | 62949-93-3 |
| Mulberrofuran A | 68978-04-1 |
| Mulberrofuran B | 79295-49-1 |
| Mulberrofuran G | 87085-00-5 |
| Mulberrofuran Q | 101383-35-1 |
| Kuwanon W | 95518-95-9 |
| Sanggenol S |  |
| Sanggenol R |  |
| Moracin P | 102841-46-3 |
| Moracin O | 123702-97-6 |
| Moracin M | 56317-21-6 |
| Kuwanon O | 89200-01-1 |
| Albanin A | 73343-42-7 |
| Albanol B | 87084-99-9 |
| Hispolon | 173933-40-9 |
| Morin | 480-16-0 |
| Mulberroside A | 102841-42-9 |
| Morusin | 62596-29-6 |
| Mulberrin | 62949-79-5 |
| kuwanon G | 75629-19-5 |
| Hymexelsin | 117842-09-8 |
| Androsin | 531-28-2 |
| Oxyresveratrol 4'-O-β-D-glucopyranoside | 863329-68-4 |
| astragalin | 480-10-4 |
| Scopolin | 531-44-2 |
| Resveratrol | 501-36-0 |
| Rutin | 153-18-4 |
| Salicylic acid | 69-72-7 |
| Oxyresveratrol 3'-O-β-D-glucopyranoside | 144525-40-6 |
| Umbelliferone | 93-35-6 |
| Chlorogenic acid | 327-97-9 |
| Multicaulisin | 286461-76-5 |
| 3'-Geranyl-3-prenyl-5,7,2',4'-tetrahydroxyflavone | 1334309-44-2 |
| Oxyresveratrol 2-O-β-D-glucopyranoside | 392274-22-5 |
| Oxyresveratrol | 29700-22-9 |
| Delphinidin chloride | 528-53-0 |
| 1-Deoxynojirimycin | 19130-96-2 |
| Coumarin 6 | 38215-36-0 |
| Methylparaben | 99-76-3 |
| (+)-δ-Tocopherol | 119-13-1 |
| Malvidin-3-O-glucoside chloride | 7228-78-6 |
| cichoriin | 531-58-8 |
| 23-Acetyl alisol C | 26575-93-9 |
| 5,6,7-Trimethoxycoumarin | 55085-47-7 |
| Morusignin L | 149733-95-9 |

**Supplementary Table S2.** The shRNA sequences were listed as below:

| ShTRIM161-1-F | CCGGGCCGTTGTTCAGCGCAAATATCTCGAGATATTTGCGCTGAACAACGGCTTTTTG |
| --- | --- |
| shTRIM16-1-R | AATTCAAAAAGCCGTTGTTCAGCGCAAATATCTCGAGATATTTGCGCTGAACAACGGC |
| shTRIM16-2-F | CCGGCCGCATCAGGTGAACATCAAACTCGAGTTTGATGTTCACCTGATGCGGTTTTTG |
| shTRIM16-2-R | AATTCAAAAACCGCATCAGGTGAACATCAAACTCGAGTTTGATGTTCACCTGATGCGG |
| shTRIM16-3-F | CCGGCTGTGTGACTTCTGCCTTGATCTCGAGATCAAGGCAGAAGTCACACAGTTTTTG |
| shTRIM16-3-R | AATTCAAAAACTGTGTGACTTCTGCCTTGATCTCGAGATCAAGGCAGAAGTCACACAG |

**Supplementary Table S3.** Primer pairs for real-time PCR:

| OPTN-F | CCAAACCTGGACACGTTTACC |
| --- | --- |
| OPTN-R | CCTCAAATCTCCCTTTCATGGC |
| GAPDH-F | CTGGGCTACACTGAGCACC |
| GAPDH-R | AAGTGGTCGTTGAGGGCAATG |

**Supplementary Table S4.** The most related gene in mass spectrometry and transcriptomics (Top 10):

| Upregulated | |
| --- | --- |
| Gene name | Fold change  (SL/DMSO) |
| OPTN | 0.35744 |
| TRIM16 | 41.29432966 |
| JUP | 4.318526494 |
| MID1 | 4.127502363 |
| HSPA5 | 1.616130596 |
| HSPA9 | 1.382768221 |
| RUVBL1 | 0.999917458 |
| EEF1A1 | 0.985387472 |
| VIM | 0.961387135 |
| HSPA1B | 0.881464754 |
| TF | 0.267194068 |

**Supplementary Table S5.** The most upregulated and downregulated gene related to autophagy and apoptosis in proteomic (Top 15):

| Upregulated | | Downregulated | |
| --- | --- | --- | --- |
| Gene name | Fold change  (SL/DMSO) | Gene name | Fold change (SL/DMSO) |
| TRIM16 | 41.29432966 | OPTN | 0.35744 |
| SESN2 | 16.29883686 | TMBIM6 | 0.39643986 |
| SGPP1 | 15.70372018 | ELAPOR1 | 0.398469473 |
| STING1 | 11.47455514 | TLK2 | 0.429676996 |
| MET | 7.667359187 | MAP1LC3A | 0.431624816 |
| IRAK4 | 7.557897267 | SOGA1 | 0.432669543 |
| EFEMP1 | 7.488051889 | FOXK1 | 0.440670733 |
| HLA-DPB1 | 5.937347039 | PIK3CB | 0.451860413 |
| SQSTM1 | 4.40395048 | ATG9A | 0.463977641 |
| WDR41 | 3.338557732 | FNBP1L | 0.516484481 |
| WIPI1 | 3.287649899 | RB1CC1 | 0.551001282 |
| MAPK15 | 3.037788505 | FBXO7 | 0.56095291 |
| BNIP1 | 2.569943757 | STAT3 | 0.594029197 |
| CALCOCO2 | 2.534951818 | EI24 | 0.62586662 |
| MAP1LC3B | 2.423312372 | HTT | 0.629253522 |

**Supplementary Table S6. Summary of Sanggenol L pharmacokinetic parameters**

| **0.083-24h** |  |  |  |
| --- | --- | --- | --- |
| PK parameters | | Unit | Mean |
| Cl_obs | | mL/min/kg | 297.9 |
| T1/2 | | h | 3.9 |
| C0 | | ng/mL | 898.3 |
| AUClast | | h*ng/mL | 828.2 |
| AUCInf | | h*ng/mL | 839.2 |
| AUC_%Extrap_obs | | % | 1.3 |
| MRTInf_obs | | h | 4.3 |
| AUClast/D | | h*mg/mL | 55.2 |
| Vss_obs | | L/kg | 77.2 |

**S****upplementary Table S7. Liposome nanomaterials particle size, PDI, Zeta potential**

|  | Intensity Size(nm) | PDI | Zeta Potential (mV) |
| --- | --- | --- | --- |
| Lipo | 180.50±1.45 | 0.104±0.01 | -36.7±0.57 |
| Lipo-SL | 181.48±2.37 | 0.180±0.02 | -38.9±0.62 |
| Lipo-SL-IR780-Biotin-HA | 182.38±2.49 | 0.137±0.02 | -40.6±0.37 |
